# Exosome circ-CBLB promotes M1 macrophage polarization in rheumatoid arthritis through the TLR3/TRAF3 signaling axis

**DOI:** 10.3389/fimmu.2025.1627389

**Published:** 2025-07-17

**Authors:** Mengyu Zhang, Lei Wan, Xiaojun Zhang, Siyu Wang, Feng Li, Dawei Yan

**Affiliations:** ^1^ Anhui University of Traditional Chinese Medicine First Clinical Medical College, Hefei, Anhui, China; ^2^ First Affiliated Hospital of Anhui University of Traditional Chinese Medicine, Hefei, Anhui, China; ^3^ Anhui University of Traditional Chinese Medicine Academic Affairs Office, Hefei, Anhui, China

**Keywords:** CBLB, TLR (Toll-Like Receptors), rheumatoid arthritis, macrophage-cell, circRNA, TRAF3, immune inflammation

## Abstract

**Background:**

Rheumatoid arthritis (RA) is a chronic autoimmune condition characterized by persistent inflammation of the joint’s synovial membrane. This inflammation leads to the degradation of joint cartilage and bone, resulting in joint dysfunction and deformity. Early symptoms of RA are often subtle, complicating timely diagnosis. Identifying potential markers for RA is therefore critical.

**Purpose and study design:**

This study aimed to explore the role of circular RNA CBLB (circ-CBLB) in RA by examining its influence on the Toll-like receptor 3/TNF receptor-associated factor 3 (TLR3/TRAF3) signaling axis and its effects on macrophage polarization through exosomes.

**Results:**

We found that exosomes may contribute to macrophage polarization, as shown through exosome uptake assays and flow cytometry. Clinical data reveal low expression levels of circ-CBLB in rheumatoid arthritis patients, correlating negatively with immunoinflammatory indices. Overexpression of circ-CBLB was found to inhibit M1 macrophage polarization. Further, binding between circ-CBLB and TLR3 was confirmed using RNA Immunoprecipitation, RNA pulldown, Western blot analysis, immunofluorescence, and quantitative reverse transcription polymerase chain reaction (qRT-PCR) techniques. Inhibiting circ-CBLB or TLR3 demonstrated that the effects on macrophage polarization could be counteracted by introducing inhibitors or inducers for M2 macrophage polarization, underscoring the significant role of exosomal circ-CBLB in RA.

**Conclusion:**

Exosomal circ-CBLB plays a crucial role in inhibiting the TLR3/TRAF3 signaling pathway, thereby reducing M1 macrophage polarization in RA patients. These findings enhance our understanding of pathophysiology of RA and offer novel insights and methods for its diagnosis and treatment.

## 1 Introduction

Rheumatoid Arthritis (RA) is a common autoimmune disease characterized by symptoms such as joint pain, swelling, stiffness, and deformity, and it can also affect multiple organ systems including the cardiovascular and respiratory systems, and the eyes, significantly impairing patient quality of life (1–4). The chronic nature of RA necessitates long-term treatment and management, contributing to the complexity of its treatment. The causes of rheumatoid arthritis are not completely understood, but are believed to involve a combination of genetic factors, environmental influences, and immune system (5, 6). If not treated early, RA can lead to irreversible joint deformity and functional loss, highlighting the importance of prompt diagnosis and intervention (7, 8). Although advancements have been made in the diagnostic and therapeutic approaches for RA, the precise underlying mechanisms remain elusive, necessitating continued research. This study seeks to delve deeper into the pathophysiological processes of RA, aiming to provide new theoretical insights and potential therapeutic avenues for its clinical management.

Exosomes are nanosized vesicles characterized by bilayer phospholipid membranes that encase proteins, lipids, DNA, and a diverse array of nucleic acids, including mRNA and microRNA (9–11). Exosomes act as carriers of signaling molecules, delivering their encapsulated cargo to recipient cells, thereby modulating the function and behavior of these cells (12). This mode of cell-to-cell communication plays a crucial role in various physiological and pathological processes, such as immune responses, neural interactions, and the metastasis of diseases (13–15). Furthermore, exosomes are increasingly recognized for their potential diagnostic and therapeutic applications in immune-related disorders (16).

circ-CBLB, derived from a circular RNA variant of the CBLB gene, belongs to the category of non-coding RNAs and exhibits unique biological properties (17, 18). Its encoded products play roles in a range of biological processes, including pathways related to immunoregulation and tumor suppression (19). The circular structure of circ-CBLB enhances its stability within the cell, enabling it to perform specific functions and potentially serving as a diagnostic marker. In our preliminary research, high-throughput sequencing and Gene Ontology (GO) analysis identified circCBLB as a key player in the RA inflammatory response. Additionally, we found that overexpression of cicCBLB reduced cytokine secretion by M1-type macrophages (20). In oncology, circ-CBLB has been shown to significantly influence regulatory mechanisms by impacting the cell cycle, triggering apoptosis, and facilitating cellular migration, all of which contribute to the regulation of tumor growth and infiltration (21–23). Importantly, circ-CBLB also plays a crucial role in the immune system, particularly in the activation and functioning of T lymphocytes, and its altered expression levels can directly affect immune response efficacy, a factor of considerable importance in immune-related diseases like systemic lupus erythematosus (SLE) and RA (24–29). However, the investigation of circ-CBLB in RA remains in its early stages, and its precise impact on this chronic inflammatory condition requires further detailed study. Enhancing our understanding of circ-CBLB in the context of RA could uncover new aspects of the disease and lead to the development of novel diagnostic and therapeutic approaches.

Recent studies have highlighted that the progression and manifestation of RA involve intricate regulatory networks associated with several signaling pathways, including the Mitogen-Activated Protein Kinase (MAPK), Janus Kinase (JAK), Nuclear Factor kappa-light-chain-enhancer of activated B cells (NF-κB), and Toll-Like Receptor (TLR) pathway (30–35). These mechanisms are crucial for understanding the pathophysiology of RA and could lead to breakthroughs in early diagnosis, tailored treatments, and the creation of novel therapeutics. In this study, we explored the role of the exosomal circ-CBLB/Toll-like receptor 3 (TLR3)/TNF receptor-associated factor 3 (TRAF3) axis in modulating the immune-inflammatory response in RA. This study addressed several aspects, namely, determination of whether exosomal circ-CBLB undergoes transfer to macrophages and influences M1/M2 polarization, verification of the molecular mechanism underlying the interaction between circ-CBLB and TLR3/TRAF3 signaling in macrophages, and evaluation of the effect of this pathway on the secretion of inflammatory cytokines. Our findings indicate that circ-CBLB, a circular RNA present in exosomes, plays a significant role in the immune-inflammatory processes of RA. Through both *in vitro* and *in vivo* studies, It was observed that exosomal circ-CBLB promoted M1 macrophage polarization through TLR3/TRAF3 pathway, and overexpression of circ-CBLB inhibited expression of TLR3 pathway components, thus blocking M1 macrophage polarization and alleviating the immune inflammatory response in RA.

## 2 Materials and methods

### 2.1 Clinical sample

This study adhered to the diagnostic criteria for RA set forth in 2010 by the American College of Rheumatology (ACR) and the European League Against Rheumatism (EULAR). It involved 50 RA patients hospitalized at the Department of Rheumatology at the First Hospital Affiliated with the Anhui University of Traditional Chinese Medicine (AUTCM). Peripheral blood samples were collected from these patients. The assays conducted included measurements of rheumatoid factor (RF), anti-cyclic citrullinated peptide antibody (CCP), erythrocyte sedimentation rate (ESR), C-reactive protein (CRP), and the markers for M1 and M2 macrophages, CD80 and CD163, respectively, along with the levels of circ-CBLB. All samples were obtained following approval from the Pharmacological Review Committee of the First Affiliated Hospital of Anhui University of Traditional Chinese Medicine (approval number 2023AH-52, approval date July 27, 2023). The study adhered to the principles of the Declaration of Helsinki. Informed consent was obtained from all individual participants included in the study.

### 2.2 Co-culture experiments

RA-FLSs were obtained from Shanghai Fuhang Biotechnology Co. and were identified by short tandem repeat (STR) analysis. The isolated cells were cultured in Dulbecco’s Modified Eagle Medium (DMEM) supplemented with 10% fetal bovine serum (FBS) and 1% penicillin-streptomycin. THP-1 monocytes were originally isolated from peripheral blood mononuclear cells (PBMC). The cells were seeded at 1*10^6 in 6-well culture plates and cultured overnight. M0 induction was performed by treating the cells with 100 ng/mLof PMA for 72 h (36). Macrophages were co-cultured with synoviocytes by Transwell co-culture chambers and co-cultured with the induced M0 for 48 h.

### 2.3 Cell transfection

RA synoviocytes were enzymatically digested and seeded at a density of 1*10^6 cells per well in 6-well plates. Transfection was conducted the following morning when the cells reached about 70% confluence. The tube containing the siRNA(General Biol Co.) in dry powder form was centrifuged at 1000 rpm for 1 minute, and 125 µl of DEPC water was added to rehydrate the siRNA. Each 100 pmol siRNA and 100 pmol the negative control (NC) were dissolved in 250 µl of serum-free medium, while 10 µl of Lipo8000™ transfection reagent was mixed with 500 µl of serum-free medium. These solutions were combined, mixed thoroughly, and left at room temperature for 5 minutes. Subsequently, the transfection mixture was divided, with 10 µl of Lipo8000™ added to both the siRNA and NC tubes, mixed well, and left to incubate at room temperature for 20 minutes. The 6-well plates were washed 2–3 times with serum-free medium before the transfection mixture (500 µl) was added. The plates were gently agitated for 1–2 minutes, the volume was adjusted to 2 ml with serum-free medium, and the plates were placed in the incubator for 4 hours. After this period, the medium was replaced with the original complete medium, and the cells were incubated for an additional 48 hours. Cells were then harvested, and the expression of target genes was quantitatively analyzed using reverse transcription-quantitative polymerase chain reaction.

### 2.4 qRT-PCR

RNA was extracted from the cells using TRIzol reagent, which was then utilized for reverse transcription and amplification processes for further analysis. Specific genes were amplified using the qRT-PCR technique, and the amplified products were analyzed with a fluorescence quantitative PCR instrument. For relative quantitative analysis, the 2-^ΔΔ^Ct method was employed, using β-actin gene expression as an internal control. The primer sequences for each assay are detailed in **Table 1**.

**Table 1:** Primers used for each assay.

Gene	Amplicon size (bp)	Forward primer (5’→3’)	Reverse primer (5’→3’)
β-actin	96	CCCTGGAGAAGAGCTACGAG	GGAAGGAAGGCTGGAAGAGT
circ-CBLB	133	TCAGCTTCCTCATGTTCAGGT	TGCTAACGGACCAGTACACTT
TLR3	117	GCCATGAAGTTGCTGACTGC	TGGCGGCTGGTAATCTTCTG
TRAF3	86	GACCGCGAGAACTCCTCTTT	CTTTAGCGGCGGGTTAGTCT
CD80	182	GGGAAATGTCGCCTCTCTGAA	TCCTGGGTCTCCAAAGGTTG
CD86	84	CAGCCAAAATGGATCCCCAG	GACTGAAGTTAGCAGAGAGCAG

### 2.5 Enzyme-Linked Immunosorbent Assay

The concentrations of The levels of interleukin 10 (IL-10, catalog no. RX103064H), tumor necrosis factor-alpha (TNF-α, catalog no. RX104793H), interleukin 6 (IL-6, catalog no. RX106126H), and interleukin 13 (IL-13, catalog no. RX104055H) were quantified using ELISA kits obtained from Quanzhou Ruixin Biotech Co.

### 2.6 Western blot analysis

To each well of a 6-well plate, 100 µl of RIPA cell lysate (containing 1 mM PMSF) was added and the mixture was lysed on ice for 30 minutes. The lysate was then centrifuged at 12,000 rpm for 15 minutes, and the supernatant was collected. Protein concentrations were measured using an Enhanced BCA Protein Assay Kit (Beyotime Biotechnology, P0010S). A 5X SDS-PAGE Protein Sampling Buffer was added to the 30-μg protein samples in a 1:4 ratio to adequately denature the proteins. The samples were then loaded onto a gel for electrophoresis, followed by transfer to a PVDF membrane (Millipore, IPVH00010). The PVDF membrane was blocked with 5% skim milk powder at room temperature for 2 hours. Blocked membranes were incubated with a series of primary antibodies, including anti-TLR3 (FabGennix, TLR-301AP, 1:500), anti-TRAF3 (Affinity, AF5380, 1:1000), anti-CD80 (Affinity, DF7682, 1:1000), and anti-CD86 (ZENBIO, 380350, 1:1000), also at room temperature. After the primary antibody incubation was completed, the membranes were washed and then incubated with horseradish peroxidase (HRP)-labeled secondary antibodies for 2 hours at room temperature. The blot was detected using an enhanced chemiluminescence kit (Beyotime). Protein blotting results were semi-quantitatively analyzed using ImageJ software with β-actin as an internal reference.

### 2.7 RNA-binding protein immunoprecipitation assay

An RNA pull-down kit (Bersinbio Guang zhou, Bes5102) was used for RNA pull-down. First, cells were collected and rinsed with 4 ml of PBS, then lysed using polysome lysis buffer with added 1.7 mL protease and RNase inhibitors. Twenty microliters of DNase and 8.5 μL of DNase salt stock were added to the supernatant, followed by incubation at 25°C for 1 hour. This was followed by the addition of 40 μL of agarose beads, 8.5 μL of EDTA, 3.4 μL of EGTA, and 17 μL of DTT to the protein sample, with gentle rotation at 4°C for 30 minutes. RNA was isolated through centrifugation, followed by immunoprecipitation utilizing protein A/G magnetic beads; specific antibodies and control IgG were incorporated, and the mixture was incubated with the cell lysate overnight. Following this, the magnetic beads were washed, and RNA was eluted using elution buffer, with the subsequent addition of 60 μL of protein elution buffer and 0.6 μLDTT. Finally, RNA was extracted with TRIzol. After several precipitation and washing steps, the RNA was dissolved in RNase-free water and analyzed via qRT-PCR.

### 2.8 Isolation of exosomes

Exosomes were isolated using ultracentrifugation to ensure their purity for downstream mechanistic studies, as the presence of contaminating cellular debris could confound the polarization results. Detection of both CD63 and CD81 was used to confirm the presence of exosomes in the preparation, while calnexin (endoplasmic reticulum marker) and GM130 (Golgi marker) were included to rule out contamination by intracellular organelles. Exosome isolation kit (Beibei Bio, product number 084001) is used for exosome isolation. Cell supernatant were collected in centrifuge tubes and centrifuged at 5000 rpm for 10 min. The supernatants were collected and filtered through 0.22-μm membranes to remove cellular debris and larger particles. Add the supernatant to a total volume of one-third EDTA-phenol (EP) solution, incubation at 0-4 °C with slow mixing for 1 h, and centrifugation at 12–000 rpm for 15 min at 4°C, with retention of the precipitate. A volume of 500-1000 μl of PBS buffer was added with blowing of the precipitate for 3–4 min, incubation in a water bath at 37 °C for 20 min to promote dissolution, and further blowing for 2–3 min for resuspension to obtain a greater exosome yield. The exosomes were stored at -80°C for further analysis. A transmission electron microscope (HT7700, Hitachi, Japan) was used for evaluation of exosome sizes and morphology. The particle sizes and concentration distributions of the exosomes were assessed using nanoparticle tracking analysis.

### 2.9 PKH26 labeling of exosomes

PKH26 fluorescent dye from the PKH26 Red Fluorescent Cell Membrane Staining Kit(Solarbio life sciences, D0030) was used to label purified exosomes. The exosome solution was first diluted with 0.5 ml of diluted CPBS. The kit provides all the necessary solvents for the staining process, including Dilution C, which enhances the solubility of the dye during the staining process, improving the staining efficiency while maintaining the viability of the exosomes. It is isotonic with mammalian cells and contains no detergents, organic solvents, physiological saline, or buffer salts. Subsequently, 0.5 ml of Dilution C and 4 µl of PKH26 dye were added, thoroughly mixed, and incubated at room temperature for 4 minutes to ensure complete binding of the dye to the exosomes. After the incubation, to remove any unbound dye, the labeled exosomes were washed through centrifugation at 100,000 g for 1 hour. Following fixation and treatment of the cells, the uptake of labeled exosomes was examined using a Olympus CKX53 inverted fluorescence microscope, which detected the red fluorescent signal.

### 2.10 Flow cytometry

The treated cells were harvested following standard procedures. First, the cells were washed twice with ice-cold phosphate-buffered saline (PBS) to remove any residues of the culture medium. To block non-specific binding, the cell suspension was incubated with 10% normal goat serum in PBS for 15 minutes at room temperature. Detection was performed using an Agilent NovoCyte flow cytometer. To gate the cell population of interest, forward scatter (FSC) and side scatter (SSC) were used to exclude debris and dead cells. The fluorescence intensities of CD80 and CD206 was measured, and the percentage of positive cells was calculated. The acquired flow cytometry images were analyzed and plotted using NovoExpress software.

### 2.11 Statistical analysis

Statistical analysis was conducted using programs like GraphPad Prism. Mann-Whitney U tests were used for comparing two sets of data. For the comparison of multiple groups of data, we used one-way analysis of variance (ANOVA), Tukey’s test was used for pairwise comparison between groups, with differences where *p*<0.05 deemed statistically significant.

## 3 Results

### 3.1 Discovery and characterization of exosomes in synoviocytes from RA patients

**Figure 1:**
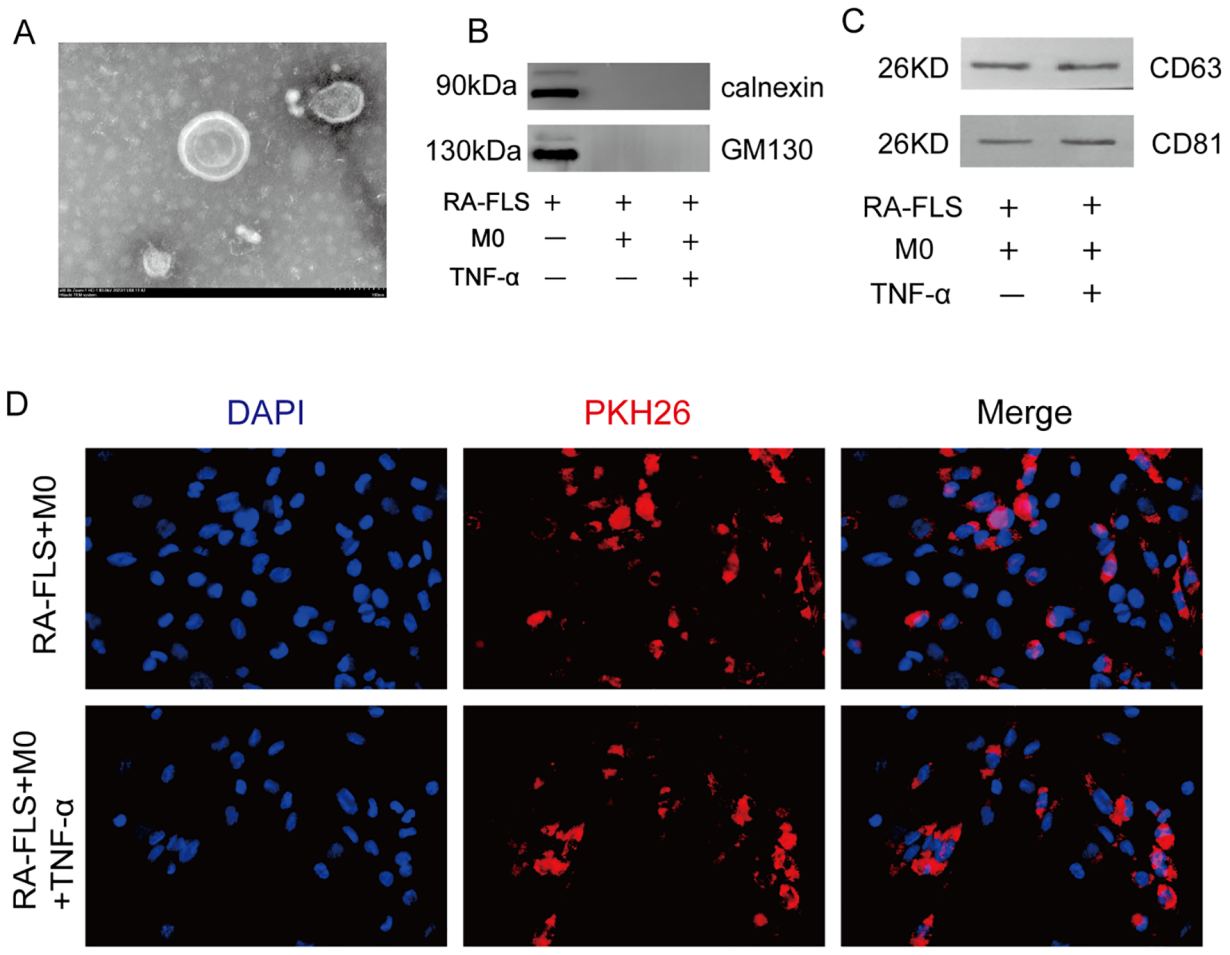
Identification and uptake of exosomes. **(A)** Visualization of exosomes in synoviocytes. **(B)** Immunoblotting analysis of the presence of calnexin and GM130 in the different experimental groups. **(C)** Immunoblotting assay results for the exosomal protein markers CD63 and CD81. **(D)** Experiment of macrophages phagocytosing exosomes and comparison of exosome uptake across different cell groups. DAPI, a blue fluorescent dye, is used to label the nuclei, and PKH26, a red fluorescent dye, is used to label the exosomes.

In a prior study by our group, we observed that circ-CBLB was downregulated in RA exosomes, suggesting its potential as a biomarker for RA (37). To confirm these findings, exosomes were first isolated, and subsequently verified, from the cell supernatants. Under transmission electron microscopy, these exosomes displayed a distinct bilayer membrane (**Figure 1A**). Their size ranged from 30 to 150 nm. The presence of the endoplasmic reticulum transmembrane protein calnexin and the Golgi matrix protein 130 (GM130) was detected to assess potential contamination. The results showed that neither calnexin nor GM130 was detected in the co-culture system, indicating that there was no intracellular protein contamination in the exosome samples (**Figure 1B**). Subsequently, we detected the exosomal protein markers CD63 and CD81 and found that both the control group (RA-FLS+M0) and the model group (RA-FLS+M0+TNF-α) of the co-culture system exhibited high expression levels of these markers (**Figure 1C**), confirming the successful extraction of exosomes. Additionally, we examined the uptake of these exosomes by macrophages using PKH26-labeled exosomes in uptake experiments and observed increased uptake in the macrophages of the Model group (**Figure 1D**). This finding indicates a potential link between exosome uptake by macrophages and macrophage polarization, establishing the feasibility of exosomal circ-CBLB transfer and providing a mechanistic basis for subsequent polarization assays.

### 3.2 Effect of exosomes on macrophage polarization

**Figure 2:**
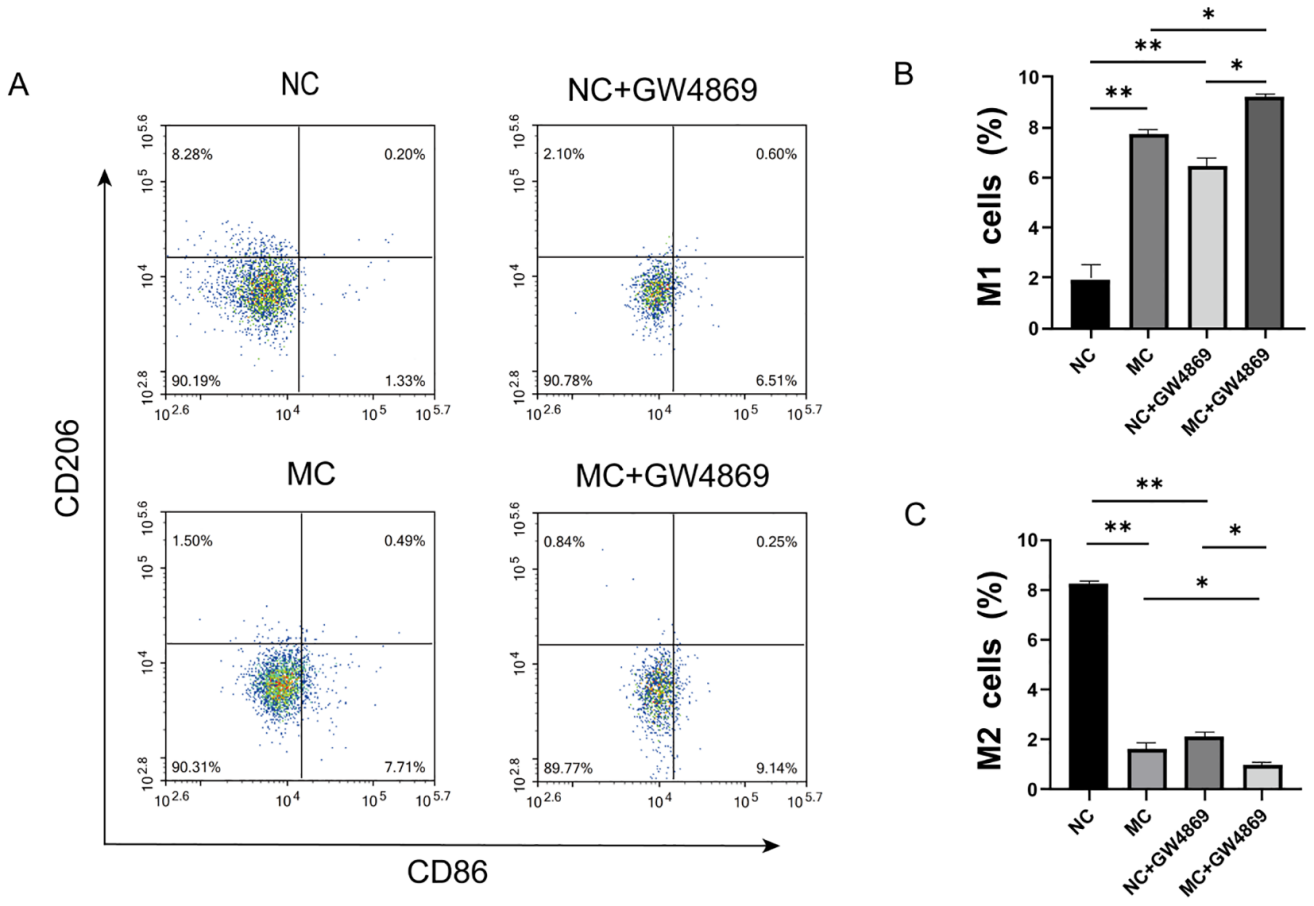
Effect of exosomes on macrophage polarization. **(A)** Flow cytometry profiles of CD86 and CD206. **(B)** Proportion of CD86 macrophages across different groups. **(C)** Proportion of CD206 macrophages across different groups. NC=Normal Control represents RA-FLS+M0, and MG=Model Group represents RA-FLS+M0+TNF-α. *p<0.05, **p<0.01.

Macrophage polarization was analyzed through co-culture of synoviocytes and macrophages, with the expression levels of the M1 macrophage marker CD86 and the M2 macrophage marker CD206 assessed via flow cytometry. We observed an increase in CD86 expression and a decrease in CD206 expression in the MC group (**Figure 2A**). After adding the exosome inhibitor GW4869 to the NC and MC groups, the expression of the M1 marker CD86 was found to be higher in the MC+GW4869 group compared to the MC group (*p*<0.05) (**Figure 2B**), while the expression of the M2 marker CD206 was lower in the MC+GW4869 group compared to the MC group (*p*<0.05) (**Figure 2C**). These results further support the hypothesis that exosomes play a role in macrophage polarization.

### 3.3 circ-CBLB was associated with RA immune-inflammatory response

**Figure 3:**
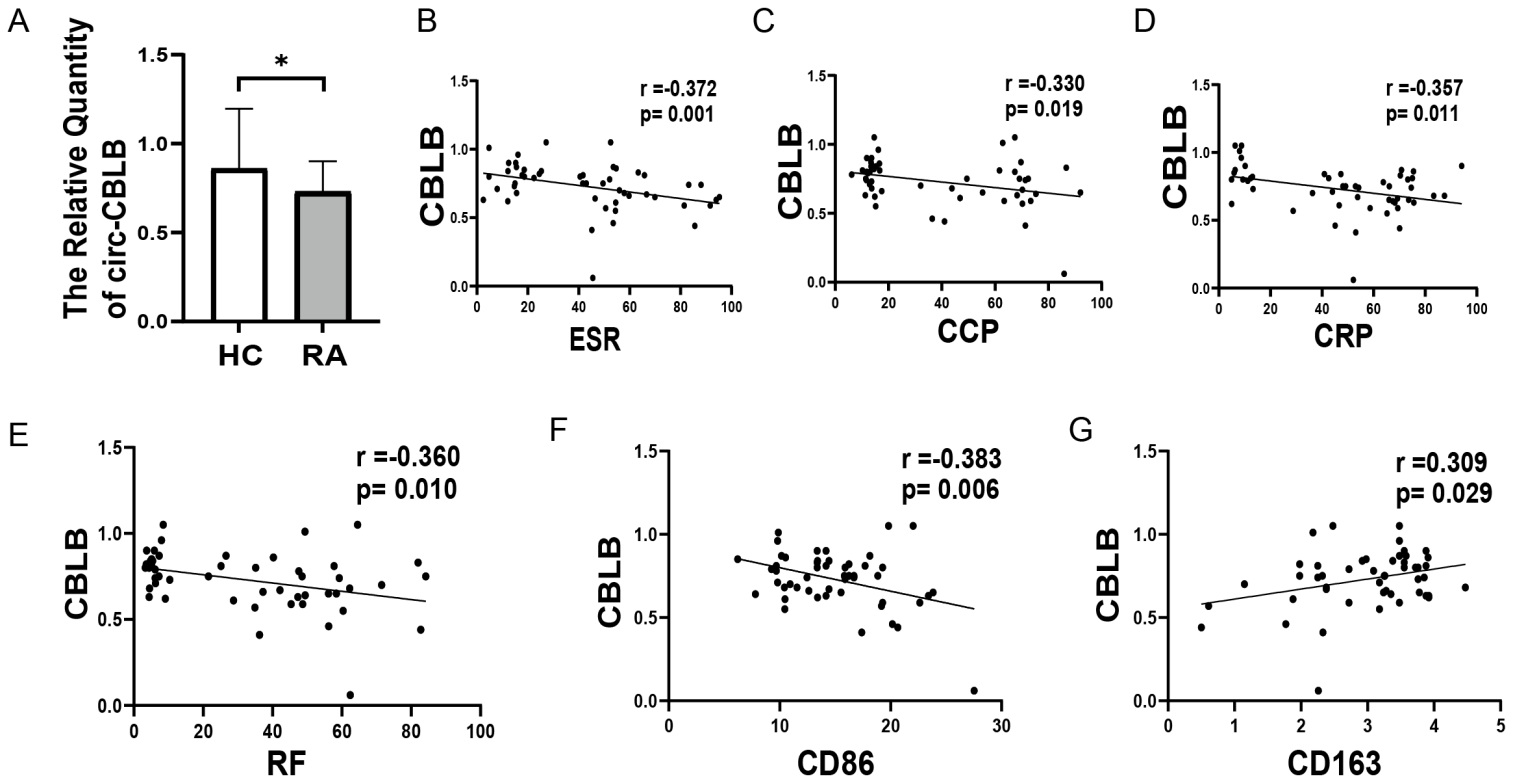
Association of circ-CBLB with immune inflammation in RA patients. **(A)** Levels of circ-CBLB in healthy individuals compared to RA patients. **(B–E)** Correlation of circ-CBLB with immune inflammation markers ESR, CRP, RF, and CCP in RA. **(F, G)** Association of circ-CBLB with macrophage polarization markers CD80 (M1) and CD163 (M2). *p<0.05, indicating significant differences compared to the HC group. HC=Healthy control represents healthy individuals, and RA represents patients with rheumatoid arthritis.

To explore the relationship between circ-CBLB and RA inflammation, we conducted clinical validation with a cohort consisting of 50 healthy individuals and 50 RA patients. The clinical characteristics of the subjects are shown in **Table 2**. We measured the levels of circ-CBLB in both groups and discovered that it was significantly lower in RA patients compared to healthy individuals (*p*<0.05) (**Figure 3A**), confirming that circ-CBLB is downregulated in RA. We also assessed indicators of immune inflammation in RA patients, such as ESR, CRP, RF, and CCP, and analyzed their correlation with circ-CBLB. The findings indicated a negative correlation between circ-CBLB and each of these indicators (**Figures 3B–E**). Furthermore, we evaluated the levels of the M1 macrophage marker CD86 and the M2 macrophage marker CD163 and examined their relationships with circ-CBLB. The results revealed a negative correlation with CD86 and a positive correlation with CD163 (**Figures 3F, G**).

**Table 2:** Clinical characteristics of HC and RA subjects.

Parameters	HC (n = 50)	RA (n = 50)	t/U/χ^2^	p
Age (years)	54.18 ± 5.60	54.0 ± 9.2	0.837	0.405
Gender (n/%)				
Male	15 (30.00)	12 (24.00)	0.556	0.456
Female	35 (70.00)	38 (76.00)		
Disease Duration (years)	–	11.0(8.0,14.0)	–	–
Treatment Status (n/%)	–		–	–
Methotrexate	–	30 (60.00)	–	–
Methotrexate + Folic acid	–	15 (30.00)	–	–
Leflunomide	–	5 (10.00)	–	–
DAS28 score	–	5.7(5.3,6.1)	–	–
circ-CBLB	0.856 ± 0.337	0.74 ± 0.142	-2.086	0.037

### 3.4 Inhibition of macrophage polarization by circ-CBLB overexpression

**Figure 4:**
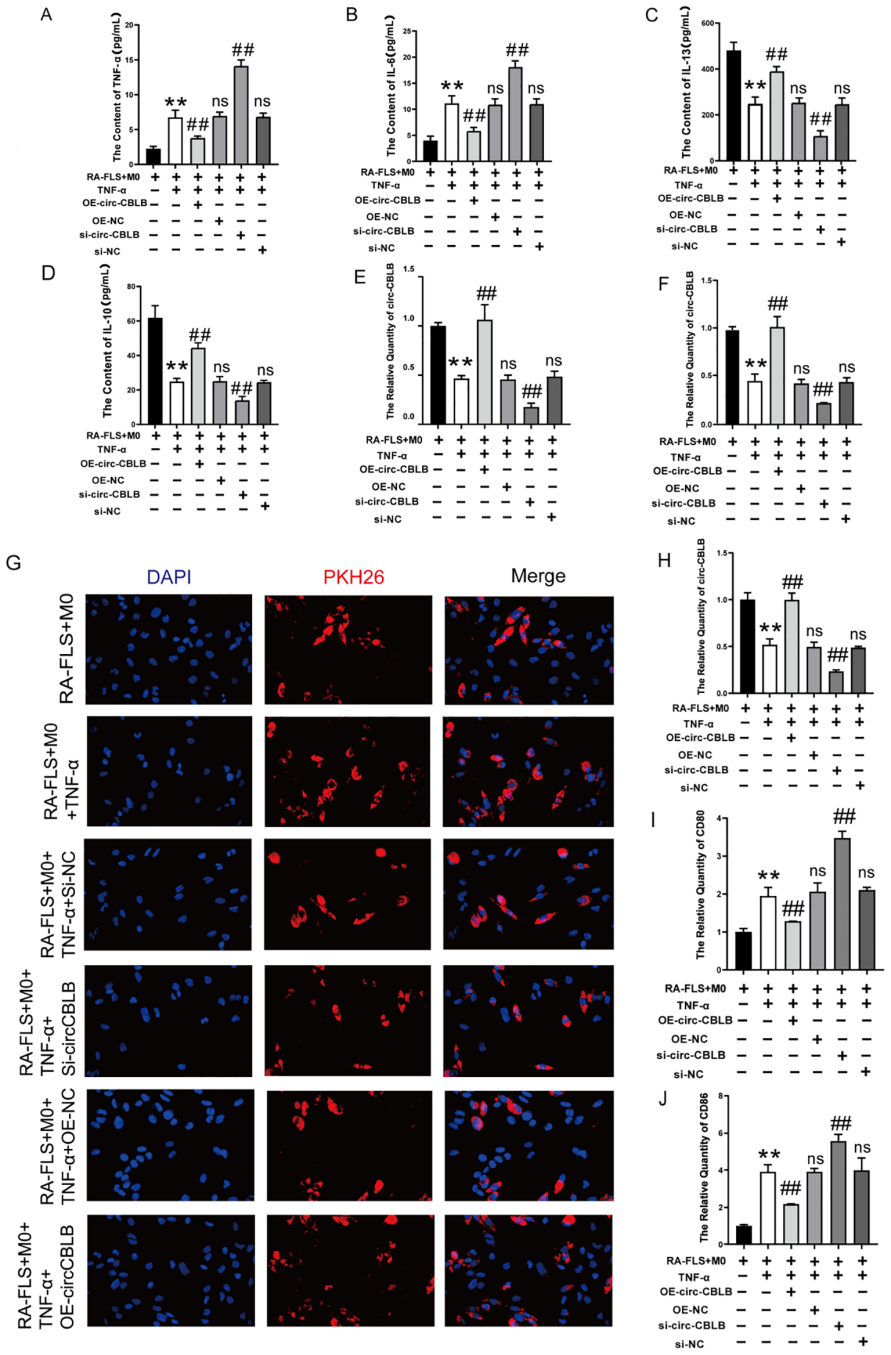
Effect of overexpression of circ-CBLB versus knockdown of circ-CBLB on the co-culture system. **(A–D)** Quantification of TNF-α, IL-6, IL-13, and IL-10 levels in a co-culture system of macrophages with RA-FLS; **(E)** qRT-PCR results showing circ-CBLB levels in synoviocytes from rheumatoid arthritis patients; **(F)** qRT-PCR results for circ-CBLB in exosomes; **(G)** Exosome uptake assay results; **(H)** qRT-PCR results for circ-CBLB in macrophages; **(I, J)** Measurement of M1 macrophage markers CD80 and CD86 in macrophages(n=3). OE-NC=OE-circ-CBLB-NC, si-NC=si-circ-CBLB-NC. **p<0.01, indicates significant differences compared to the RA-FLS+M0 group. ^##^ *p*<0.01, indicates significant differences compared to the RA-FLS+M0+TNF-α group.

To investigate the relationship between circ-CBLB and macrophage polarization, we measured the levels of TNF-α, IL-6, IL-13, and IL-10 in co-cultured macrophages and RA-FLS cells. We observed that in the RA-FLS+M0 group, TNF-α and IL-6 were present at low levels, while IL-13 and IL-10 were elevated. In contrast, the addition of TNF-α to the RA-FLS+M0 group led to increased levels of TNF-α and IL-6, and decreased levels of IL-13 and IL-10. circ-CBLB knockdown resulted in significantly increased TNF-α and IL-6 levels in the co-culture system compared to the RA-FLS+M0+TNF-α group, and notably reduced IL-13 and IL-10 levels compared to the model group. Conversely, overexpression of circ-CBLB led to significantly reduced TNF-α and IL-6 levels and increased IL-13 and IL-10 levels compared to the model group, indicating that circ-CBLB overexpression may suppress the expression of pro-inflammatory factors (**Figures 4A–D**).

RNA was extracted from RA-FLS cells, exosomes, and macrophages using Trizol extraction, and the expression of circ-CBLB was analyzed via qRT-PCR. We found that circ-CBLB expression was significantly reduced in the synoviocytes of rheumatoid arthritis patients (*p*<0.01) (**Figure 4E**), and similarly in exosomes (*p*<0.01) (**Figure 4F**). Additionally, overexpression of circ-CBLB led to increased exosomal uptake and higher circ-CBLB levels in macrophages (*p*<0.01) (**Figures 4G, H**), indicating a potential role of circ-CBLB in influencing macrophage polarization through exosomes.

Further analysis was conducted on the impact of modulating circ-CBLB on macrophage polarization, specifically examining the expression of the M1 macrophage markers CD80 and CD86. Results showed that both markers were significantly increased upon circ-CBLB knockdown (*p*<0.01) and decreased with circ-CBLB overexpression (*p*<0.01), as depicted in **Figures 4I, J**. These findings suggest that overexpressing circ-CBLB may inhibit M1 macrophage polarization.

### 3.5 TLR3 was identified as a potential target of circ-CBLB

**Figure 5:**
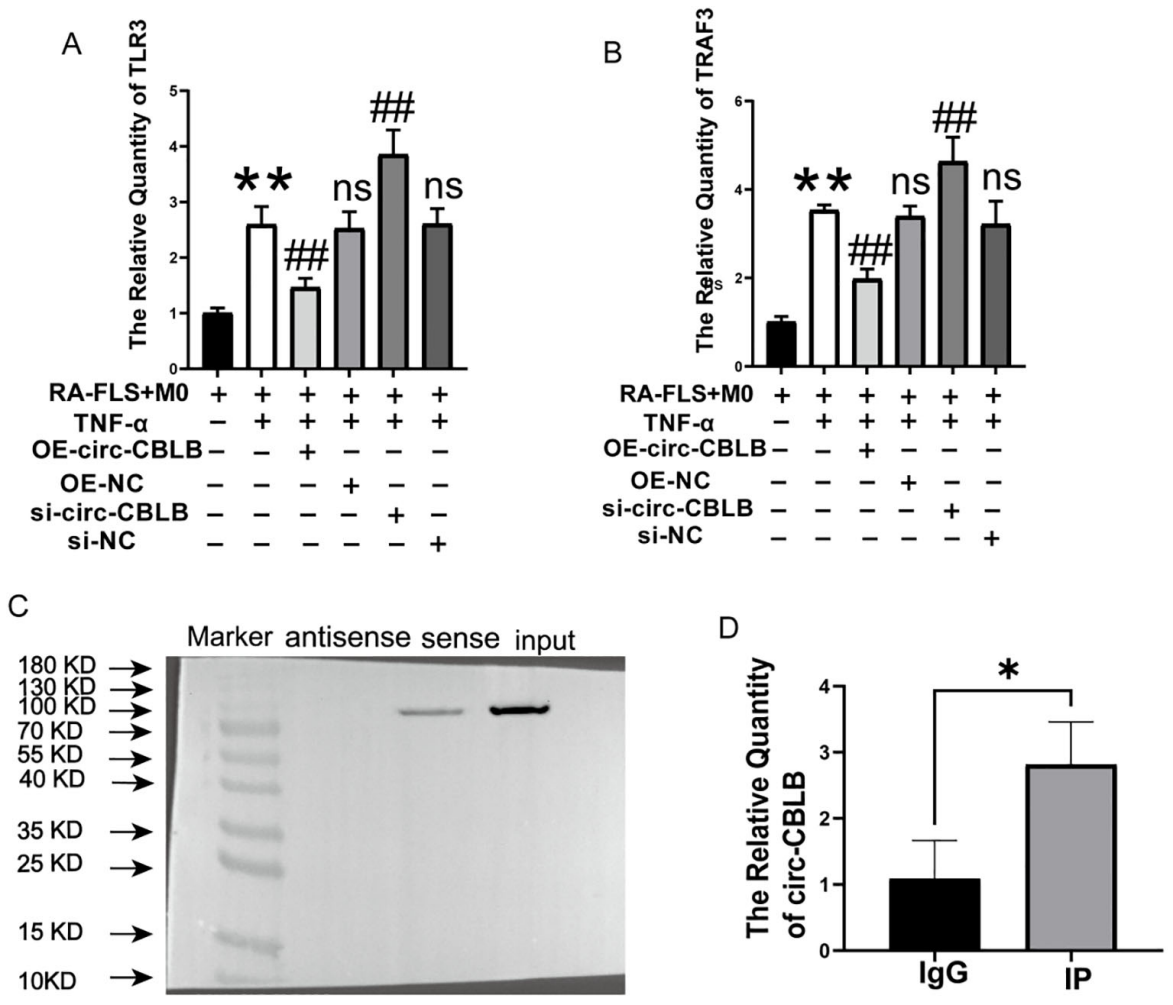
Validation of circ-CBLB targeting. **(A, B)** Levels of TLR3 and TRAF3 in the co-culture system. **(C)** Western blot verification of circ-CBLB’s binding to TLR3. **(D)** RNA immunoprecipitation (RIP) experiments demonstrating circ-CBLB enrichment in cells. **p<0.01 indicates significant differences compared to the RA-FLS+M0 group. OE-NC=OE-circ-CBLB-NC, si-NC=si-circ-CBLB-NC. ^##^ *p*<0.01 indicates significant differences compared to the RA-FLS+M0+TNF-α group. *p<0.05 indicates significant differences compared to the IgG control group.

Given the significant effect of circ-CBLB on macrophage polarization, bioinformatics analyses were then used to explore the downstream effects of circ-CBLB by prediction of its target genes. The catRAPID database (http://service.tartaglialab.com/page/catrapid_group) was used to identify interactions between specific mRNAs and proteins, The predicted downstream target proteins of circ-CBLB included TLR3, SIDT1, CPSF2, and NUP153. Many studies have shown that TLR3 plays a crucial role in the pathogenesis of RA (38, 39). TLR3 is highly expressed in the synovial tissues and peripheral blood mononuclear cells of RA patients, with its expression level closely related to disease activity (40). Considering the role of TLR3 in RA-associated inflammation, the potential interactions between circular RNAs and TLR signaling pathways, and the strong potential for binding between circ-CBLB and TLR3 mRNA predicted by the catRAPID database, we ultimately selected TLR3 as the candidate target. The targeting relationship between circ-CBLB and TLR3 was thus investigated, with verification using RNA pull-down and RIP assays.TLR3, a pattern recognition receptor, recognizes pathogenic double-stranded RNA (dsRNA) associated molecular patterns (PAMPs) and activates downstream signaling to trigger an immune response. TRAF3, a member of the Toll/IL-1 receptor family and a cellular signaling molecule, binds to TLR3 and participates in its signaling pathway. We then measured the levels of TLR3 and TRAF3 in the co-culture system and found that their expression was significantly increased upon the knockdown of circ-CBLB (*p*<0.01) and decreased with the overexpression of circ-CBLB (*p*<0.01), as illustrated in **Figures 5A, B**. This pattern was consistent with the expression changes of CD80 and CD86 in the RA co-culture system, suggesting that circ-CBLB modulates M1 macrophage polarization through the TLR3/TRAF3 signaling pathway, thereby exerting anti-inflammatory and immunoregulatory effects. This was further supported by RNA immunoprecipitation (RIP) experiments, where circ-CBLB was found to be enriched with TLR3 (**Figures 5C, D**), confirming our hypothesis.

### 3.6 Circ-CBLB downregulated TLR3/TRAF3 signaling pathway to inhibit M1 macrophage polarization

**Figure 6:**
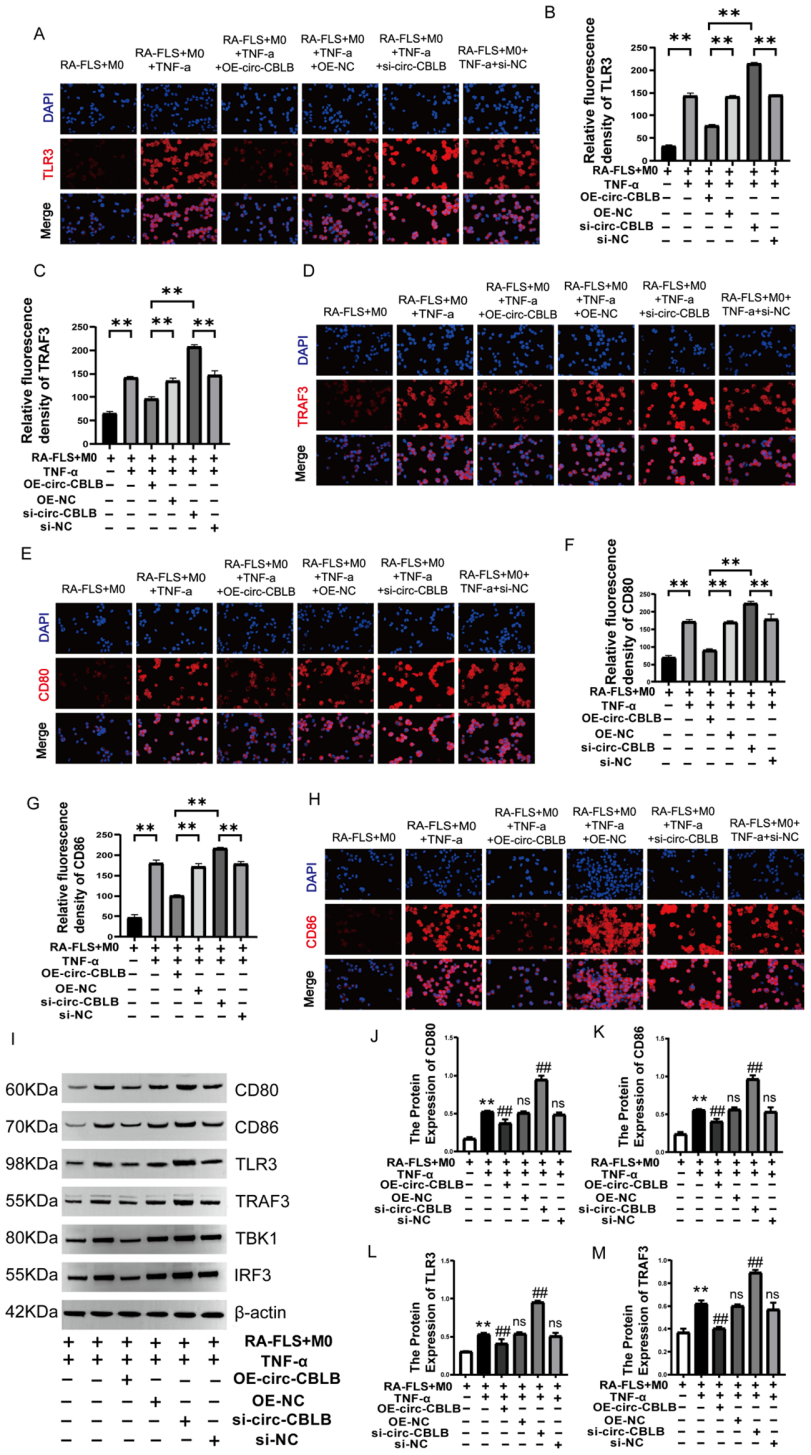
Impact of circ-CBLB manipulation on TLR3/TRAF3 expression. **(A, B)** Immunofluorescence detection of TLR3 expression;**p<0.01; **(C, D)** Immunofluorescence detection of TRAF3 expression;**p<0.01; **(E, F)** Immunofluorescence detection of CD80 expression; **p<0.01; **(G, H)** Immunofluorescence detection of CD86 expression; **p<0.01; **(I)** Western blot analysis of TLR3, TRAF3, CD80, CD86, TBK1, and IRF3 proteins in the co-culture system. **(J–M)** Protein expression of TLR3, TRAF3, CD80 and CD86. DAPI was used to stain the nucleus(n=3). OE-NC=OE-circ-CBLB-NC, si-NC=si-circ-CBLB-NC. **p<0.01, compared to the RA-FLS+M0 group; ^##^ *p*<0.01, compared to the RA-FLS+M0+TNF-α group.

Immunofluorescence was employed to assess the expression and localization of TLR3 (**Figures 6A, B**), TRAF3 (**Figures 6C, D**), CD80 (**Figures 6E, F**), and CD86 (**Figures 6G, H**) proteins in macrophages. The fluorescence intensity for TLR3, TRAF3, CD80, and CD86 significantly decreased following overexpression of circ-CBLB (*p*<0.01). In addition, Western blotting was used to determine the protein expression levels of TLR3, TRAF3, CD80, CD86, TBK1, and IRF3 in the co-culture system. The results showed that after overexpression of circ-CBLB, the protein expression of TLR3, TRAF3, TBK1, IRF3, CD80, and CD86 was reduced, but were increased after inhibition of circ-CBLB (**Figure 6I**). Quantitative analysis of the protein levels of TLR3, TRAF3, CD80 and CD86 was performed, and the results were consistent with those of Western blotting. Following the overexpression of circ-CBLB, the protein expression levels of TLR3, TRAF3, CD80 and CD86 were increased (*p*<0.01) (**Figures 6J–M**). These results suggest that overexpressing circ-CBLB leads to a downregulation of TLR3/TRAF3, thereby inhibiting the polarization of macrophages toward the M1 phenotype.

### 3.7 Effects on M1 macrophages after TLR3 Inhibition can be reversed by circ-CBLB inhibitors

**Figure 7:**
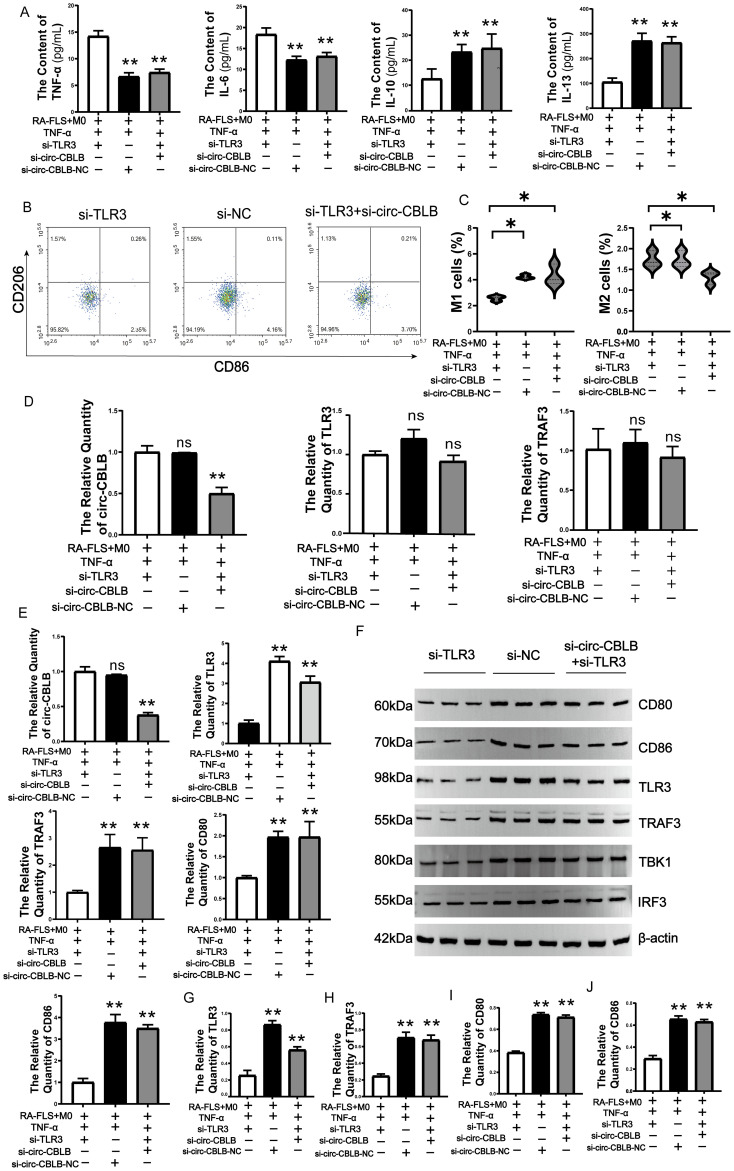
Effects of TLR3 inhibition on circ-CBLB, TLR3, TRAF3, CD80, CD86. **(A)** Expression of TNF-a, IL-6, IL-13, IL-10 in co-cultured cells. **(B, C)** Variations in the expression of M1 and M2 macrophage markers; **(D)** qRT-PCR analysis of circ-CBLB, TLR3, and TRAF3 expression in exosomes. **(E)** qRT-PCR assessment of circ-CBLB, TLR3, and TRAF3 in macrophages. **(F)** Immunoblotting to evaluate the expression levels of TLR3, TRAF3, CD80, CD86, TBK1 and IRF3 proteins in the co-culture system(n=3). **(G–J)** Protein expression of TLR3, TRAF3, CD80 and CD86. *p<0.05, **p<0.01 indicate significant differences compared to the RA-FLS+M0+TNF-α+si-TLR3 group.

To further investigate the role of circ-CBLB in this signaling pathway, we conducted experiments where TLR3 overexpression was inhibited alongside circ-CBLB. Subsequently, we assessed the levels of TNF-α, IL-6, IL-13, and IL-10 in the co-culture system. The results indicated that suppressing TLR3 expression significantly increased the levels of TNF-α and IL-6 (*p*<0.01) while reducing IL-10 and IL-13 levels (*p*<0.01). Further inhibition of circ-CBLB led to a decrease in TNF-α and IL-6 levels (*p*<0.01) and an increase in IL-10 and IL-13 levels (*p*<0.01) (**Figure 7A**). This suggests that TLR3 inhibition enhances the secretion of pro-inflammatory factors, but additional suppression of circ-CBLB can reverse this effect.

We also analyzed total macrophage protein to confirm these findings, observing that TLR3 inhibition reduced the percentage of M1 macrophages and increased that of M2 macrophages. Additional inhibition of circ-CBLB resulted in an increase in M1 macrophage markers and a decrease in M2 markers (*p*<0.05) (**Figures 7B, C**).The expression of circ-CBLB, TLR3, and TRAF3 in exosomes was further examined. It was found that the expression of exosomal circ-CBLB, TLR3, and TRAF3 did not show significant changes when TLR3 alone was inhibited. However, when circ-CBLB was additionally inhibited, its expression decreased, with no significant changes in TLR3 and TRAF3 expression observed (**Figure 7D**). In macrophages, the inhibition of TLR3 leads to a significant decrease in the expression of TLR3, TRAF3, and CD80. When circ-CBLB is inhibited simultaneously with TLR3, the contents of TLR3, TRAF3, CD80, and CD86 increase. (*p*<0.01) (**Figure 7E**).

The Western blotting results showed that inhibition of TLR3 led to a decrease in the protein expression of TLR3, TRAF3, TBK1, IRF3, CD80, and CD86 in the co-culture system. Furthermore, when circ-CBLB was inhibited, the protein expression of TLR3, TRAF3, TBK1, IRF3, CD80, and CD86 increased instead (**Figure 7F**). The results of the protein quantification of TLR3, TRAF3, CD80, and CD86 also showed the same situation (**Figures 7G–J**).These findings indicate that circ-CBLB is involved in the TLR3/TRAF3 signaling pathway, influencing macrophage polarization. Inhibiting TLR3 reduced the levels of CD80 and CD86, but this effect was reversed when both TLR3 and circ-CBLB were inhibited.

### 3.8 Effects on macrophage polarization after inhibition of circ-CBLB can be reversed by M2 macrophage polarization inducers

**Figure 8:**
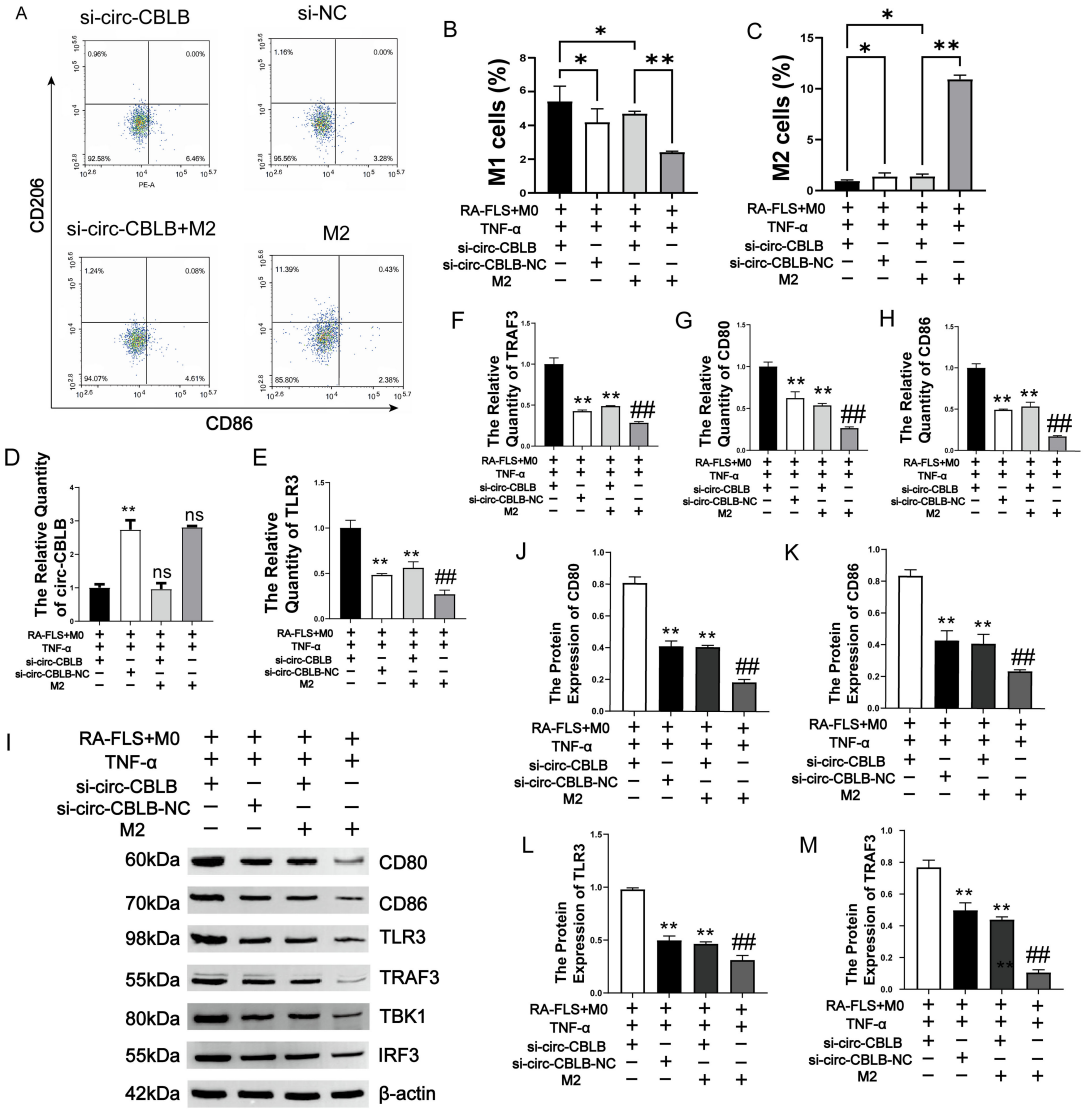
Impact of M2 Polarization Induction on circ-CBLB, TLR3, TRAF3, CD80, and CD86. **(A)** Detection of macrophage polarization via flow cytometry. **(B, C)** Ratios of M1 to M2 macrophages; **(D)** Levels of circ-CBLB in exosomes. **(E–H)** Expression of TLR3, TRAF3, CD80, and CD86 in macrophages. **(I)** Use of immunoblotting to assess TLR3, TRAF3, CD80, CD86, TBK1 and IRF protein levels in macrophages. **(J–M)** Quantitative analysis of TLR3, TRAF3, CD80, and CD86 proteins in macrophages using immunoblotting(n=3). *p<0.01, **p<0.01 indicate significant differences compared to the RA-FLS+M0+TNF-α+si-circ-CBLB group; ^##^ *p*<0.01, indicates significant differences compared to the RA-FLS+M0+TNF-α+si-circ-CBLB+M2 group.

Upon inhibiting circ-CBLB in the RA-FLS+M0+TNF-α group, while simultaneously adding an M2 macrophage polarization inducer into the co-culture, we monitored various indices related to macrophage polarization. We observed an increase in the percentage of M1 macrophages following the inhibition of circ-CBLB (*p*<0.01). However, when the inhibition of circ-CBLB was combined with the addition of the M2 macrophage polarization inducer, there was a decrease in the percentage of M1 macrophages (*p*<0.01). Using only the M2 macrophage polarization inducer resulted in a decrease in M1 macrophage percentage (*p*<0.01), and the subsequent inhibition of circ-CBLB along with the M2 inducer led to an increase in M1 macrophage percentage (*p*<0.01) (**Figures 8A–C**). The results indicate that circ-CBLB and TLR3 are negatively correlated. When circ-CBLB was inhibited, there was a significant increase in the expression of TLR3, TRAF3, CD80, and CD86 (*p*<0.01). Additionally, combining the inhibition of circ-CBLB with the addition of an M2 macrophage polarization inducer led to a decrease in the expression levels of TLR3, TRAF3, CD80, and CD86 (*p*<0.01). Conversely, the expression levels of TLR3, TRAF3, CD80, and CD86 were found to be higher (*p*<0.01) when compared to those in the M2 group alone (**Figures 8D–H**).

**Figure 9:**
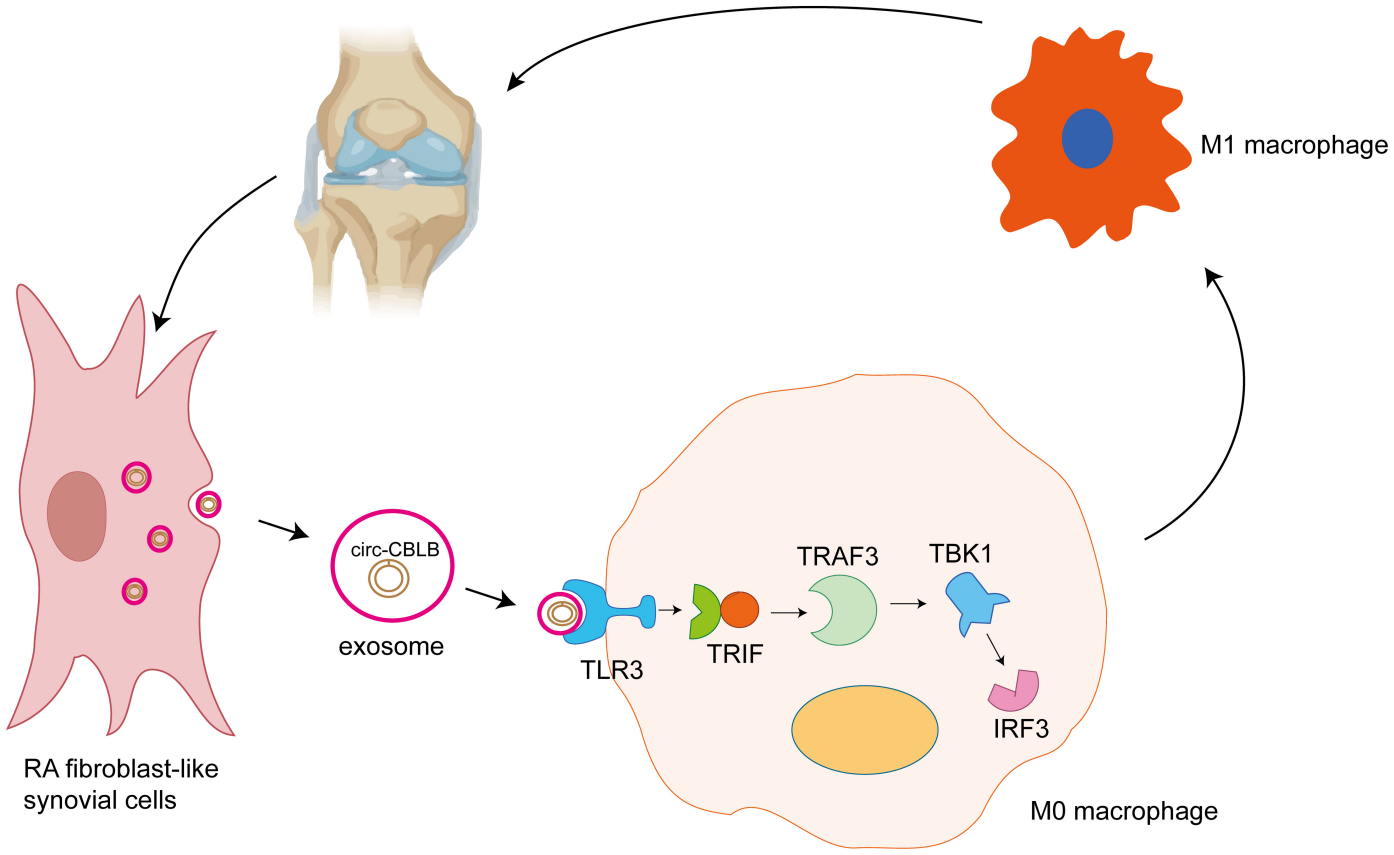
Exosomal circ-CBLB promotes M1 macrophage polarization in rheumatoid arthritis through the TLR3-TRAF3 signaling axis. RA-FLS secrete exosomes (small circular vesicles), which contain circ-CBLB (the highlighted circ-RNA molecule within the exosome). Exosomes are important in intercellular communication, transporting a variety of biomolecules between cells. The exosomes containing circ-CBLB are taken up by M0 macrophages, undifferentiated macrophages that can undergo polarization to the pro-inflammatory M1 phenotype or anti-inflammatory M2 phenotype depending on the stimuli they receive. When taken up by the M0 macrophages, circ-CBLB activates the TLR3 pathway. TLR3 is a pattern recognition receptor that senses the presence of viral double-stranded RNA and other nucleic acid-containing pathogens. In this case, it recognizes the exosomal circ-CBLB. Activation of TLR3 leads to the recruitment and activation of TRIF, a key adaptor molecule in the TLR3 signaling cascade. TRIF then activates TRAF3, an important downstream component of the TLR pathway. TRAF3 subsequently activates TBK1 and IRF3. Activation of the TLR3-TRAF3 signaling axis ultimately drives polarization of M0 macrophages to the M1 phenotype. M1 macrophages are characterized by their pro-inflammatory function, and secrete cytokines such as TNF-α, IL-6, and other inflammatory mediators. These cytokines contribute to the chronic inflammation and joint destruction seen in rheumatoid arthritis. Further secretion of cytokines by the M1 macrophages can stimulate the RA-FLS, creating a positive feedback loop that exacerbates the inflammatory state in the joint tissue. This continuous cycle of inflammation and cell-cell communication is central to the pathogenesis of rheumatoid arthritis.

The results of WB assay for TLR3, TRAF3, TBK1, IRF3, CD80, and CD86 proteins are shown in **Figure 8I**. The TLR3, TRAF3, TBK, IRF3, CD80, and CD86 protein contents in the si-circ-CBLB group were significantly higher than that in the si-NC group. Additionally, in the si-circ-CBLB+M2 group, the levels of these proteins were significantly reduced compared to the si-circ-CBLB group alone, but remained higher than those observed in the M2 group alone. The quantitative results of TLR3, TRAF3, CD80 and CD86 are shown in **Figures 8J–M**. These findings further indicate that circ-CBLB plays a role in macrophage polarization through the TLR3 pathway, and that its inhibitory effects can be mitigated by M2 macrophage polarization inducers. The proposed mechanism is shown in **Figure 9**.

## 4 Discussion

RA is an autoimmune disorder marked by persistent inflammation of the synovial lining (41). Central to the pathogenesis of RA is the abnormal proliferation and reduced apoptosis of FLS, which are key contributors (2, 42–44). These cells demonstrate tumor-like proliferative traits, are invasive, and draw significant numbers of inflammatory cells into the joints. This influx results in the production of substantial inflammatory mediators and chemokines, aggravating the progression of the disease (45, 46).This study aimed to determine whether exosomal circ-CBLB from RA synovial fibroblasts modulates macrophage polarization *via* the TLR3/TRAF3 signaling axis.

Exosomes have gained notable attention in the context of RA due to their critical role in cell-to-cell communication and disease pathology (11, 16). These small membranous vesicles are secreted by cells and carry molecules like circ-CBLB, facilitating intercellular communication (47, 48). In RA, exosomes are primarily derived from synovial fibroblasts and immune cells, contributing to the perpetuation of the inflammatory response (12). Previous studies have shown that exosomes can influence macrophage differentiation, prompting us to further explore their impact on macrophage polarization (49, 50). In RA, extracellular vesicles (EVs), particularly exosomes, have emerged as key mediators of the immunopathogenesis of the disease. Most of these exosomes originate from leukocytes and synovial cells, and carry pro-inflammatory molecules that perpetuate the chronic inflammatory cascade characteristic of RA. Notably, proteins with post-translational modifications (PTMs) have been identified in EVs from RA patients, and certain subsets of these vesicles have been found to be capable of activating dendritic cells. The modified proteins transported by these vesicles may exert immunostimulatory effects, while increased levels of specific proteins and RNAs within EVs can promote the development of inflammatory arthritis and RA progression (16). Within the broader context of immune dysregulation and its impact on autoimmune diseases, studies investigating X-chromosome aneuploidies, such as Klinefelter syndrome (47, XXY), have contributed significantly to our understanding of RA (51). In individuals with X-chromosome aneuploidies, genetic and molecular dysregulation leads to systemic autoimmunity. As the X chromosome harbors numerous genes involved in immune regulation, the presence of extra copies disrupts the normal balance of nucleic acid and protein expression. This results in aberrations of immune cell function and the production of non-organ-specific autoantibodies, aligning with observations in autoimmune diseases such as RA. Mechanistically, the present research focused on the role of exosomal circ-CBLB and TLR3-associated pathways in RA-associated macrophage polarization. However, it is important to contextualize these findings within the broader immunomodulatory functions of EVs. Pattern recognition receptors (PRRs), including TLR3, are key players in the detection of a wide variety of molecular patterns and can be activated by components within EVs. Macrophage polarization, a critical process in RA-associated inflammation, is influenced not only by circ-CBLB but also by other immunogenic factors carried within EVs. Immune-stimulatory proteins within EVs may act synergistically with circ-CBLB to promote an M1-biased macrophage phenotype, thereby fueling the inflammatory milieu in RA joints. In this study, we observed increased exosome uptake in macrophages in the Model group through exosome uptake assays, aligning with earlier findings. Using flow cytometry, we noted a significant increase in M1 macrophage markers following the addition of exosome inhibitors, indicating that exosomes might play a role in M1 macrophage polarization. circ-CBLB is a non-coding RNA with important functions and has been shown to be involved in the pathogenesis of RA through a variety of mechanisms (18, 52).

Our previous research showed that circ-CBLB inhibited the proliferation of RA-FLS, promoted their apoptosis, and modulated cytokine levels by increasing the production of anti-inflammatory cytokines, such as IL-4 and IL-10, while decreasing the levels of pro-inflammatory cytokines, including IL-6 and TNF-α (20, 37). In this study, we observed that circ-CBLB expression levels were significantly reduced in FLS from RA patients. Furthermore, circ-CBLB expression was negatively correlated with immunoinflammation-related indices in RA, including RF, ESR, CCP, and CRP, suggesting its potential association with disease activity and inflammation severity. We also examined the relationship between circ-CBLB and RA-related inflammatory cytokines. Knockdown of circ-CBLB resulted in increased levels of TNF-α and IL-6, along with decreased levels of IL-13 and IL-10. Conversely, overexpression of circ-CBLB reduced TNF-α and IL-6 levels while elevating IL-13 and IL-10 levels, consistent with prior studies. Building on these findings, we further explored the role of circ-CBLB in M1 macrophage polarization. Overexpression of circ-CBLB significantly reduced the expression of M1 macrophage markers CD80 and CD86, indicating that circ-CBLB mitigated the inflammatory response in RA by modulating M1 macrophage polarization.

The TLR3 signaling pathway plays an important role in the pathogenesis of RA (38, 53–56). Previous studies have shown that TLR3 is upregulated in RA-FLS, where its activation leads to the production of interferon-β (IFN-β), a key component of the innate immune response (57). TLR3 activation typically leads to the activation of multiple downstream signaling pathways. Excessive activation of TLR3 induces the translocation of IRF3, IRF7, and NF-κB to the nucleus, leading to increased transcription of IFNα/β, promoting the interferon response and regulating the production of inflammatory cytokines (58, 59). Previous studies have indicated the potential involvement of TBK1 in inflammatory and autoimmune diseases (60), which can promote activation of the NF-κB and interferon regulatory factor 3 (IRF3) pathways, as well as the M1 polarization of macrophages (61). Once activated, TBK1 phosphorylates a series of downstream target proteins. One of the important targets is IRF3. Phosphorylation of IRF3 by TBK1 causes its dimerization and translocation into the nucleus, where it binds to specific DNA sequences in the promoters of interferon-related genes, thereby promoting the transcription of type I interferons (62). This may be related to the regulation of immune inflammation in RA by the TLR3 pathway.

Our study further revealed the regulatory role of circ-CBLB in immune modulation. We observed that circ-CBLB is significantly enriched with TLR3. Knockdown of circ-CBLB led to increased TLR3 expression, whereas overexpression of circ-CBLB reduced the expression levels of TLR3, TRAF3, and the M1 macrophage markers CD80 and CD86. Replication experiments were conducted by inhibiting TLR3 and utilizing M2 macrophage polarization inducers. Inhibition of TLR3 significantly reduced the expression of TLR3, TRAF3, CD80, and CD86. However, further inhibition of circ-CBLB in this context resulted in elevated levels of TLR3, TRAF3, CD80, and CD86 proteins. Comparatively, in the RA-FLS+M0+TNF-α+circ-CBLB group, inhibition of circ-CBLB combined with the addition of an M2 macrophage polarization inducer decreased the expression of TLR3, TRAF3, CD80, and CD86. As circ-CBLB knockdown increased TLR3 expression, and TLR3 inhibition reversed M1 polarization, we conclude that circ-CBLB exerts its anti-inflammatory effects *via* the TLR3/TRAF3 pathway. The rescue experiment with M2 inducers further verified the specificity of this mechanism. In conclusion, circ-CBLB promotes macrophage polarization toward the M1 phenotype by regulating the TLR3/TRAF3 signaling pathway, thereby exacerbating the inflammatory response in rheumatoid arthritis. Modulating circ-CBLB expression can influence the TLR3/TRAF3 pathway to exert anti-inflammatory effects.

There are some innovations in this study. We found through clinical studies that circ-CBLB was under-expressed in RA patients. Uniquely, our research demonstrated that exosomal circ-CBLB binds to the TLR3 protein, modulating the TLR3/TRAF3 signaling pathway to inhibit macrophage polarization toward the M1 phenotype. This hypothesis was substantiated through two replicate experiments, with Western blotting and immunofluorescence further confirming the pivotal role of circ-CBLB in regulating TLR3-mediated macrophage M1 polarization. Using qPCR, RIP, RNA pull-down, and response experiments, we validated a novel circ-CBLB/TLR3 regulatory axis. Our findings indicate that circ-CBLB, carried by exosomes derived from RA fibroblast-like synoviocytes, contributes to macrophage M1 polarization via the TLR3 signaling pathway, thereby intensifying the inflammatory response in RA.

This study introduces novel concepts and approaches for the clinical management of RA, providing a mechanistic framework in which exosomes derived from RA synoviocytes transfer circ-CBLB to macrophages, where it binds to TLR3 and inhibits TRAF3 signaling, thereby reducing M1 polarization. These findings, ranging from exosome characterization to pathway verification, establish a causal relationship between circ-CBLB and RA inflammation. However, there are some limitations. The cross-sectional nature of the study precludes inferences of causality or the assessment of circ-CBLB levels over time. The study also did not include long-term follow-up and was thus unable to observe the effect of circ-CBLB on disease progression over time, all limiting in-depth exploration of the molecular mechanisms by which circ-CBLB affects macrophage polarization through the TLR3/TRAF3 pathway. Therefore, in future studies, we will investigate the effects of circ-CBLB over time on disease progression as an entry point, which will enhance our understanding of the mechanism and potential clinical applications of circ-CBLB in RA therapy. While the *in vitro* data establish a mechanistic link between exosomal circ-CBLB and macrophage polarization, verification in animal models of RA is essential to confirm these effects *in vivo*. Such studies will be critical to address biodistribution, dosage, and safety considerations for potential clinical translation.

## 5 Conclusion

In summary, our results substantiate that the therapeutic impact of circ-CBLB on RA operates through the TLR3/TRAF3 signaling pathway. By inhibiting M1 macrophage polarization, circ-CBLB effectively reduces inflammation associated with RA.

## Data Availability

The datasets presented in this study can be found in online repositories. The names of the repository/repositories and accession number(s) can be found in the article/**Supplementary Material**.

## References

[1] Buchanan WW Kean CA Kean WF Rainsford KD. Rheumatoid arthritis. Inflammopharmacology. (2024) 32:3–11. doi: 10.1007/s10787-023-01221-0, PMID: 37195496

[2] Anderson RJ Cui J Weinblatt ME Solomon DH Naik C Shadick NA. Patterns of involvement of the hand joints in classical rheumatoid arthritis. J Clin Rheumatol. (2023) 29:230–4. doi: 10.1097/RHU.0000000000001971, PMID: 37158761

[3] Schattner A. The cardiovascular burden of rheumatoid arthritis-implications for treatment. Am J Med. (2023) 136:1143–6. doi: 10.1016/j.amjmed.2023.09.004, PMID: 37742851

[4] Lyu Q Ma L Liu H Wang J. Meta-analysis of risk factors for cardiovascular disease in patients with rheumatoid arthritis. Med (Baltimore). (2023) 102:e35912. doi: 10.1097/MD.0000000000035912, PMID: 37960768 PMC10637523

[5] Cheng F Zhu Y Liu X Zhang R Xia F Ge L. Analysis of the causal relationship between immune cells and rheumatoid arthritis from the perspective of genetic variation: a bidirectional two-sample Mendelian randomization study. Adv Rheumatol. (2024) 64:83. doi: 10.1186/s42358-024-00425-4, PMID: 39487558 PMC13488670

[6] Cai X Jin J Ye H Xiang X Luo L Li J. Altered serum metabolome is associated with disease activity and immune responses in rheumatoid arthritis. Clin Rheumatol. (2024) 43:3669–78. doi: 10.1007/s10067-024-07201-1, PMID: 39485556 PMC11582171

[7] Faison MN Davis AM Trotter KC. Disease-modifying drugs for adult-onset rheumatoid arthritis. JAMA. (2024) 331:1055–6. doi: 10.1001/jama.2023.26504, PMID: 38451547

[8] Madav Y Barve K Prabhakar B. Current trends in theranostics for rheumatoid arthritis. Eur J Pharm Sci. (2020) 145:105240. doi: 10.1016/j.ejps.2020.105240, PMID: 31987984

[9] Soto-Vazquez YM Genschmer KR. Impact of extracellular vesicles on the pathogenesis, diagnosis, and potential therapy in cardiopulmonary disease. Front Pharmacol. (2023) 14:1081015. doi: 10.3389/fphar.2023.1081015, PMID: 36891265 PMC9986338

[10] Abebaw D Akelew Y Adugna A Teffera ZH Tegegne BA Fenta A. Extracellular vesicles: immunomodulation, diagnosis, and promising therapeutic roles for rheumatoid arthritis. Front Immunol. (2024) 15:1499929. doi: 10.3389/fimmu.2024.1499929, PMID: 39624102 PMC11609219

[11] Tavasolian F Moghaddam AS Rohani F Abdollahi E Janzamin E Momtazi-Borojeni AA. Exosomes: Effectual players in rheumatoid arthritis. Autoimmun Rev. (2020) 19:102511. doi: 10.1016/j.autrev.2020.102511, PMID: 32171920

[12] Miao HB Wang F Lin S Chen Z. Update on the role of extracellular vesicles in rheumatoid arthritis. Expert Rev Mol Med. (2022) 24:e12. doi: 10.1017/erm.2021.33, PMID: 35297366 PMC9884793

[13] Engeroff P Vogel M. The potential of exosomes in allergy immunotherapy. Vaccines (Basel). (2022) 10(1):133. doi: 10.3390/vaccines10010133, PMID: 35062793 PMC8780385

[14] Jan AT Rahman S Sarko DK Redwan EM. Editorial: Exploring the role of exosomes in disease progression and therapeutics in neurodegeneration. Front Aging Neurosci. (2023) 15:1177063. doi: 10.3389/fnagi.2023.1177063, PMID: 37056685 PMC10086332

[15] Wu M Wang G Hu W Yao Y Yu X. Emerging roles and therapeutic value of exosomes in cancer metastasis. Mol Cancer. (2019) 18:53. doi: 10.1186/s12943-019-0964-8, PMID: 30925925 PMC6441156

[16] Riitano G Recalchi S Capozzi A Manganelli V Misasi R Garofalo T. The role of autophagy as a trigger of post-translational modifications of proteins and extracellular vesicles in the pathogenesis of rheumatoid arthritis. Int J Mol Sci. (2023) 24:12764. doi: 10.3390/ijms241612764, PMID: 37628944 PMC10454292

[17] Zhang M Xin Y. Circular RNAs: a new frontier for cancer diagnosis and therapy. J Hematol Oncol. (2018) 11:21. doi: 10.1186/s13045-018-0569-5, PMID: 29433541 PMC5809913

[18] Zheng Y Cai X Ren F Yao Y. The role of non-coding RNAs in fibroblast-like synoviocytes in rheumatoid arthritis. Int J Rheum Dis. (2024) 27:e15376. doi: 10.1111/1756-185X.15376, PMID: 39439368

[19] Ma J Huang L Gao YB Li MX Chen LL Yang L. Circ_TNFRSF21 promotes cSCC metastasis and M2 macrophage polarization via miR-214-3p/CHI3L1. J Dermatol Sci. (2023) 111:32–42. doi: 10.1016/j.jdermsci.2023.06.001, PMID: 37442735

[20] Wan L Liu J Huang C Zhu Z Li F Sun G. Role of m6A modification and novel circ_0066715/miR-486-5p/ETS1 axis in rheumatoid arthritis macrophage polarization progression. Aging (Albany NY). (2022) 14:10009–26. doi: 10.18632/aging.204439, PMID: 36541909 PMC9831719

[21] Yang S Wang P Wang S Cong A Zhang Q Shen W. miRNA-181a-5p enhances the sensitivity of cells to cisplatin in esophageal adenocarcinoma by targeting CBLB. Cancer Manag Res. (2020) 12:4981–90. doi: 10.2147/CMAR.S251264, PMID: 32612385 PMC7323973

[22] Mao W Huang X Wang L Zhang Z Liu M Li Y. Circular RNA hsa_circ_0068871 regulates FGFR3 expression and activates STAT3 by targeting miR-181a-5p to promote bladder cancer progression. J Exp Clin Cancer Res. (2019) 38:169. doi: 10.1186/s13046-019-1136-9, PMID: 30999937 PMC6472097

[23] Meng S Zhou H Feng Z Xu Z Tang Y Li P. CircRNA: functions and properties of a novel potential biomarker for cancer. Mol Cancer. (2017) 16:94. doi: 10.1186/s12943-017-0663-2, PMID: 28535767 PMC5440908

[24] Schanz O Cornez I Yajnanarayana SP David FS Peer S Gruber T. Tumor rejection in Cblb(-/-) mice depends on IL-9 and Th9 cells. J Immunother Cancer. (2021) 9(7):e002889. doi: 10.1136/jitc-2021-002889, PMID: 34272310 PMC8287598

[25] Doniz-Padilla L Martinez-Jimenez V Nino-Moreno P Abud-Mendoza C Hernandez-Castro B Gonzalez-Amaro R. Expression and function of Cbl-b in T cells from patients with systemic lupus erythematosus, and detection of the 2126 A/G Cblb gene polymorphism in the Mexican mestizo population. Lupus. (2011) 20:628–35. doi: 10.1177/0961203310394896, PMID: 21558139

[26] Luo Q Zhang L Fang L Fu B Guo Y Huang Z. Circular RNAs hsa_circ_0000479 in peripheral blood mononuclear cells as novel biomarkers for systemic lupus erythematosus. Autoimmunity. (2020) 53:167–76. doi: 10.1080/08916934.2020.1728529, PMID: 32093518

[27] Guo G Wang H Ye L Shi X Yan K Lin K. Hsa_circ_0000479 as a novel diagnostic biomarker of systemic lupus erythematosus. Front Immunol. (2019) 10:2281. doi: 10.3389/fimmu.2019.02281, PMID: 31608065 PMC6771011

[28] Li LJ Zhu ZW Zhao W Tao SS Li BZ Xu SZ. Circular RNA expression profile and potential function of hsa_circ_0045272 in systemic lupus erythematosus. Immunology. (2018) 155:137–49. doi: 10.1111/imm.12940, PMID: 29700819 PMC6099170

[29] Luo Q Zhang L Li X Fu B Guo Y Huang Z. Identification of circular RNAs hsa_circ_0044235 and hsa_circ_0068367 as novel biomarkers for systemic lupus erythematosus. Int J Mol Med. (2019) 44:1462–72. doi: 10.3892/ijmm.2019.4302, PMID: 31432107 PMC6713423

[30] Jiang H Zhang J Yu H Hou A Wang S Wang X. Anti-rheumatoid arthritis effects of Xanthii Fructus by affecting the PI3K-AKT signaling pathway based on TMT-labeled quantitative proteomics. BioMed Chromatogr. (2023) 37:e5520. doi: 10.1002/bmc.5520, PMID: 36205398

[31] Weng W Liu Y Hu Z Li Z Peng X Wang M. Macrophage extracellular traps promote tumor-like biologic behaviors of fibroblast-like synoviocytes through cGAS-mediated PI3K/Akt signaling pathway in patients with rheumatoid arthritis. J Leukoc Biol. (2024) 115:116–29. doi: 10.1093/jleuko/qiad102, PMID: 37648663

[32] Su J Li S Chen J Jian C Hu J Du H. Glycerophospholipid metabolism is involved in rheumatoid arthritis pathogenesis by regulating the IL-6/JAK signaling pathway. Biochem Biophys Res Commun. (2022) 600:130–5. doi: 10.1016/j.bbrc.2022.02.003, PMID: 35219101

[33] Shi L Zhao Y Feng C Miao F Dong L Wang T. Therapeutic effects of shaogan fuzi decoction in rheumatoid arthritis: Network pharmacology and experimental validation. Front Pharmacol. (2022) 13:967164. doi: 10.3389/fphar.2022.967164, PMID: 36059943 PMC9428562

[34] Banerjee K Chandrasekar B Sathish S Sohn H Madhavan T. Computational drug repositioning for IL6 triggered JAK3 in rheumatoid arthritis using FDA database. Mol Divers. (2024) 29:2049–61. doi: 10.21203/rs.3.rs-3791539/v1, PMID: 39141207

[35] Chen Q Li H Liu Y Zhao M. Epigenetic regulation of immune and inflammatory responses in rheumatoid arthritis. Front Immunol. (2022) 13:881191. doi: 10.3389/fimmu.2022.881191, PMID: 35479077 PMC9035598

[36] Feng D Li Y Zheng H Wang Y Deng J Liu T. IL-4-induced M2 macrophages inhibit fibrosis of endometrial stromal cells. Reprod Biol. (2024) 24:100852. doi: 10.1016/j.repbio.2023.100852, PMID: 38354656

[37] Li S Wan L Liu J Huang C Chen Y Cheng J. Circular RNA Cbl proto-oncogene B (circCBLB) inhibits proliferation, promotes apoptosis, and increases anti-inflammatory cytokine levels of fibroblast-like synoviocytes in rheumatoid arthritis. Xi Bao Yu Fen Zi Mian Yi Xue Za Zhi. (2024) 40:106–13. 38284251

[38] Razin M Abdel-Ghaffar AB Hamdy GM Abd-Elshafy DN Kamel S Bahgat MM. TLR3\TLR7 as differentially expressed markers among viral, nonviral, and autoimmune diseases in Egyptian patients. Viral Immunol. (2021) 34:607–21. doi: 10.1089/vim.2021.0006, PMID: 34342515

[39] Liu Y Mo C Luo X Li H Guo H Sun H. Activation of toll-like receptor 3 induces interleukin-1 receptor antagonist expression by activating the interferon regulatory factor 3. J Innate Immun. (2020) 12:304–20. doi: 10.1159/000504321, PMID: 31865314 PMC7383292

[40] Abdelwahab A Palosaari S Abdelwahab SA Rifaai RA El-Tahawy NF Saber EA. Differential synovial tissue expression of TLRs in seropositive and seronegative rheumatoid arthritis: A preliminary report. Autoimmunity. (2021) 54:23–34. doi: 10.1080/08916934.2020.1864729, PMID: 33377396

[41] Feng ZW Tang YC Sheng XY Wang SH Wang YB Liu ZC. Screening and identification of potential hub genes and immune cell infiltration in the synovial tissue of rheumatoid arthritis by bioinformatic approach. Heliyon. (2023) 9:e12799. doi: 10.1016/j.heliyon.2023.e12799, PMID: 36699262 PMC9868484

[42] Feng ZW Yang CF Xiao HF Yuan F Chen F Zhang B. YTHDC1 regulates the migration, invasion, proliferation, and apoptosis of rheumatoid fibroblast-like synoviocytes. Front Immunol. (2024) 15:1440398. doi: 10.3389/fimmu.2024.1440398, PMID: 39534605 PMC11554466

[43] Kemble S Croft AP. Critical role of synovial tissue-resident macrophage and fibroblast subsets in the persistence of joint inflammation. Front Immunol. (2021) 12:715894. doi: 10.3389/fimmu.2021.715894, PMID: 34539648 PMC8446662

[44] Qian H Deng C Chen S Zhang X He Y Lan J. Targeting pathogenic fibroblast-like synoviocyte subsets in rheumatoid arthritis. Arthritis Res Ther. (2024) 26:103. doi: 10.1186/s13075-024-03343-4, PMID: 38783357 PMC11112866

[45] Liu Y Gu Y Han Y Zhang Q Jiang Z Zhang X. Tumor exosomal RNAs promote lung pre-metastatic niche formation by activating alveolar epithelial TLR3 to recruit neutrophils. Cancer Cell. (2016) 30:243–56. doi: 10.1016/j.ccell.2016.06.021, PMID: 27505671

[46] Yang X Li J Xu C Zhang G Che X Yang J. Potential mechanisms of rheumatoid arthritis therapy: Focus on macrophage polarization. Int Immunopharmacol. (2024) 142:113058. doi: 10.1016/j.intimp.2024.113058, PMID: 39236455

[47] Nabariya DK Heinz A Derksen S Krauss S. Intracellular and intercellular transport of RNA organelles in CXG repeat disorders: The strength of weak ties. Front Mol Biosci. (2022) 9:1000932. doi: 10.3389/fmolb.2022.1000932, PMID: 36589236 PMC9800848

[48] Wang X Sun L Zhou Y Su QJ Li JL Ye L. Heroin Abuse and/or HIV Infection Dysregulate Plasma Exosomal miRNAs. J Neuroimmune Pharmacol. (2020) 15:400–8. doi: 10.1007/s11481-019-09892-9, PMID: 31828734 PMC7286798

[49] Hakki SS Batoon L Koh AJ Kannan R Mendoza-Reinoso V Rubin J. The effects of preosteoblast-derived exosomes on macrophages and bone in mice. J Cell Mol Med. (2024) 28:e18029. doi: 10.1111/jcmm.18029, PMID: 37929757 PMC10805488

[50] Chamberlain CS Kink JA Wildenauer LA McCaughey M Henry K Spiker AM. Exosome-educated macrophages and exosomes differentially improve ligament healing. Stem Cells. (2021) 39:55–61. doi: 10.1002/stem.3291, PMID: 33141458 PMC7821004

[51] Panimolle F Tiberti C Spaziani M Riitano G Lucania G Anzuini A. Non-organ-specific autoimmunity in adult 47,XXY Klinefelter patients and higher-grade X-chromosome aneuploidies. Clin Exp Immunol. (2021) 205:316–25. doi: 10.1111/cei.13616, PMID: 33978253 PMC8374223

[52] Chen YM Zhu Q Cai J Zhao ZJ Yao BB Zhou LM. Upregulation of T Cell Receptor Signaling Pathway Components in Gestational Diabetes Mellitus Patients: Joint Analysis of mRNA and circRNA Expression Profiles. Front Endocrinol (Lausanne). (2021) 12:774608. doi: 10.3389/fendo.2021.774608, PMID: 35046894 PMC8763273

[53] Zhou M Feng H Wang T Xu Z Gu S Li L. TLR3 as an emerging molecule facilitating pyroptosis in the context of rheumatoid arthritis: A study combined bioinformatics and experimental validation. Cytokine. (2025) 187:156875. doi: 10.1016/j.cyto.2025.156875, PMID: 39884182

[54] Karpus ON Heutinck KM Wijnker PJM Tak PP Hamann J. Triggering of the dsRNA sensors TLR3, MDA5, and RIG-I induces CD55 expression in synovial fibroblasts. PloS One. (2012) 7:e35606. doi: 10.1371/journal.pone.0035606, PMID: 22590509 PMC3349673

[55] Samarpita S Kim JY Rasool MK Kim KS. Investigation of toll-like receptor (TLR) 4 inhibitor TAK-242 as a new potential anti-rheumatoid arthritis drug. Arthritis Res Ther. (2020) 22:16. doi: 10.1186/s13075-020-2097-2, PMID: 31973752 PMC6979396

[56] Frank-Bertoncelj M Pisetsky DS Kolling C Michel BA Gay RE Jungel A. TLR3 ligand poly(I:C) exerts distinct actions in synovial fibroblasts when delivered by extracellular vesicles. Front Immunol. (2018) 9:28. doi: 10.3389/fimmu.2018.00028, PMID: 29434584 PMC5797482

[57] Plociennikowska A Frankish J Moraes T Del Prete D Kahnt F Acuna C. TLR3 activation by Zika virus stimulates inflammatory cytokine production which dampens the antiviral response induced by RIG-I-like receptors. J Virol. (2021) 95:e1020-50. doi: 10.1128/JVI.01050-20, PMID: 33658344 PMC8139665

[58] Liu S Li M Sun F Zhang J Liu F. Enhancing the immune effect of oHSV-1 therapy through TLR3 signaling in uveal melanoma. J Cancer Res Clin Oncol. (2023) 149:901–12. doi: 10.1007/s00432-022-04272-y, PMID: 36030435 PMC11797164

[59] De Oliveira RTG Cordeiro JVA Vitoriano BF de Lima Melo MM Sampaio LR de Paula Borges D. ERVs-TLR3-IRF axis is linked to myelodysplastic syndrome pathogenesis. Med Oncol. (2021) 38:27. doi: 10.1007/s12032-021-01466-1, PMID: 33594613

[60] Ouyang Z Xu J Liu T Lin S Sun Y Huang Y. STING/TBK1 regulates inflammation in macrophages and titanium particles-induced osteolysis. ACS Biomater Sci Eng. (2023) 9:3273–84. doi: 10.1021/acsbiomaterials.2c01509, PMID: 37134278

[61] Vijay K. Toll-like receptors in immunity and inflammatory diseases: Past, present, and future. Int Immunopharmacol. (2018) 59:391–412. doi: 10.1016/j.intimp.2018.03.002, PMID: 29730580 PMC7106078

[62] Chen Y Lin J Zhao Y Ma X Yi H. Toll-like receptor 3 (TLR3) regulation mechanisms and roles in antiviral innate immune responses. J Zhejiang Univ Sci B. (2021) 22:609–32. doi: 10.1631/jzus.B2000808, PMID: 34414698 PMC8377577

