# Supplementary figures and images for: Exosome circ-CBLB promotes M1 macrophage polarization in rheumatoid arthritis through the TLR3/TRAF3 signaling axis
Source: Front Immunol. 2025 Jul 17;16:1627389. doi: 10.3389/fimmu.2025.1627389 (PMC12310475; doi:10.3389/fimmu.2025.1627389)

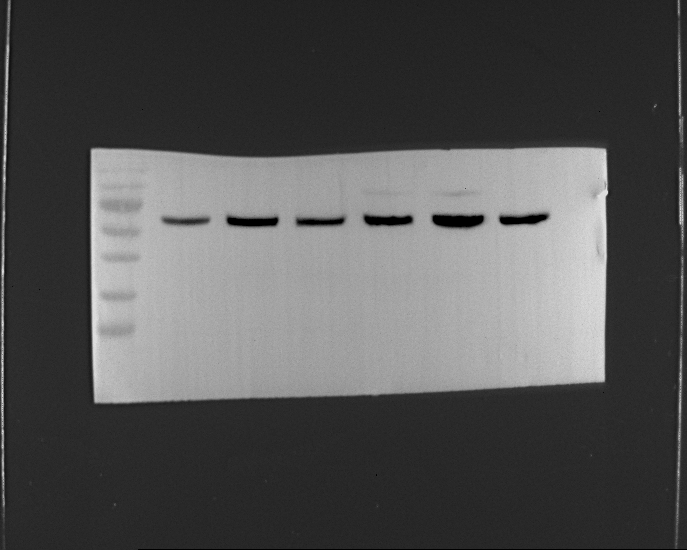

Supplement: Supplementary file 1 [file DataSheet1.zip › western blot/Fig 6/Fig6.CD80.tif]

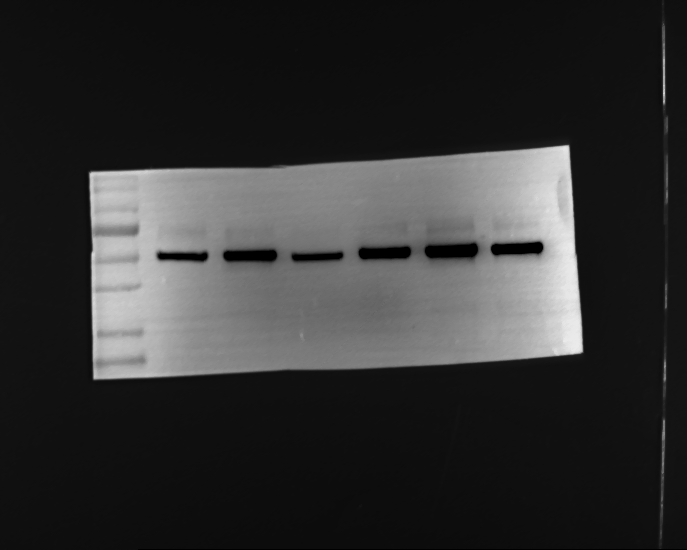

Supplement: Supplementary file 1 [file DataSheet1.zip › western blot/Fig 6/Fig6.IRF3.tif]

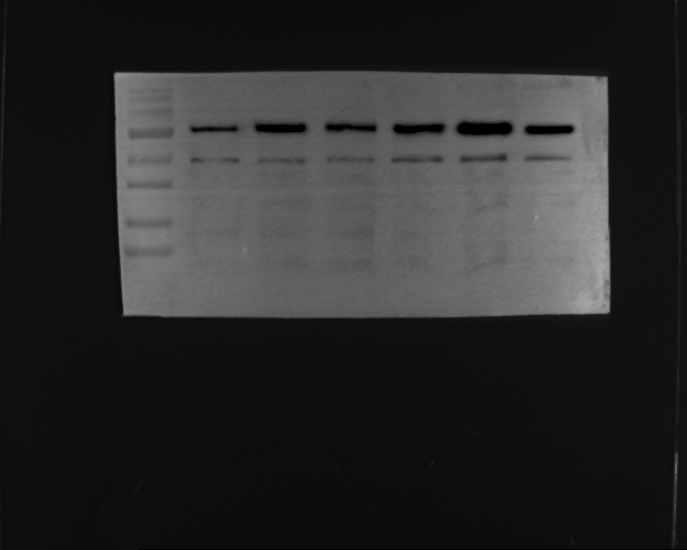

Supplement: Supplementary file 1 [file DataSheet1.zip › western blot/Fig 6/Fig6.TBK1.tif]

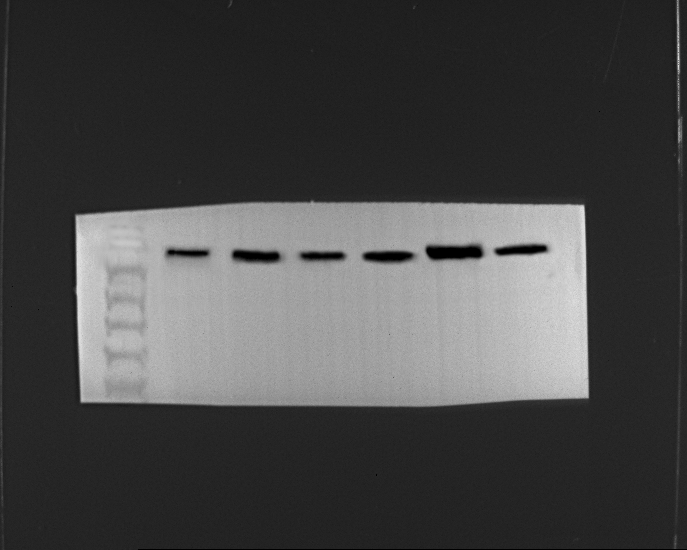

Supplement: Supplementary file 1 [file DataSheet1.zip › western blot/Fig 6/Fig6.TLR3.tif]

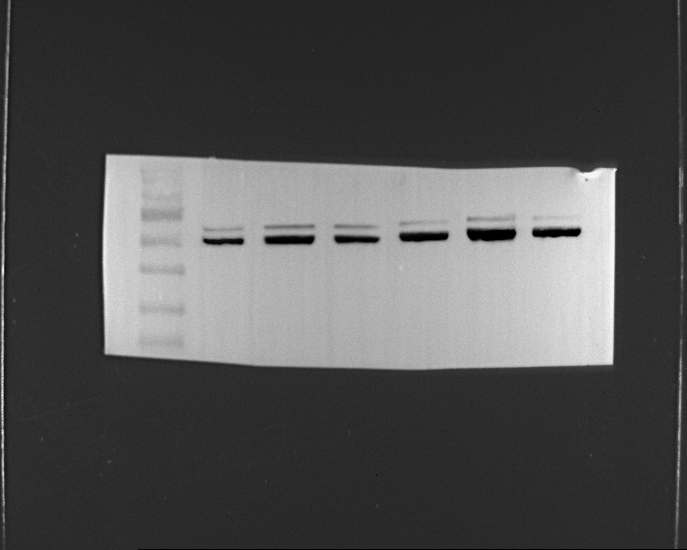

Supplement: Supplementary file 1 [file DataSheet1.zip › western blot/Fig 6/Fig6.TRAF3.tif]

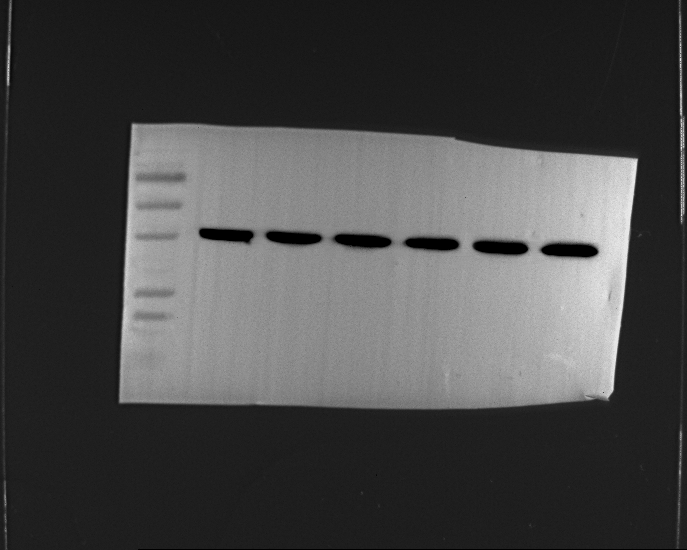

Supplement: Supplementary file 1 [file DataSheet1.zip › western blot/Fig 6/Fig6.a┬-actin.tif]

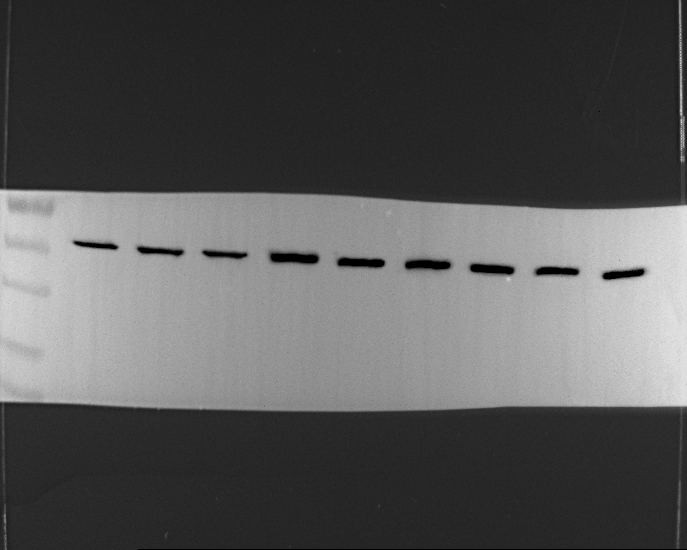

Supplement: Supplementary file 1 [file DataSheet1.zip › western blot/Fig 7/Fig7.CD80.tif]

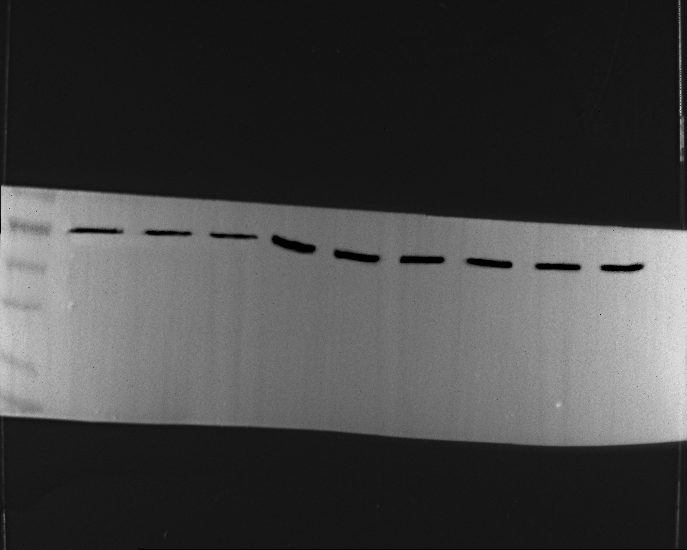

Supplement: Supplementary file 1 [file DataSheet1.zip › western blot/Fig 7/Fig7.CD86.tif]

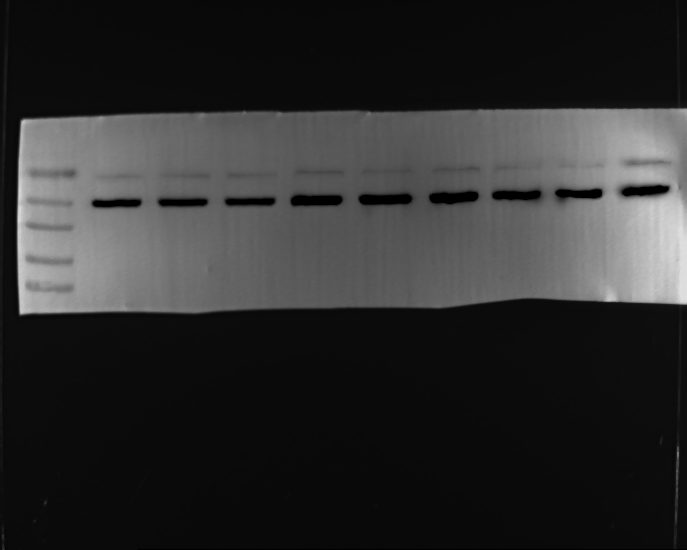

Supplement: Supplementary file 1 [file DataSheet1.zip › western blot/Fig 7/Fig7.IRF3.tif]

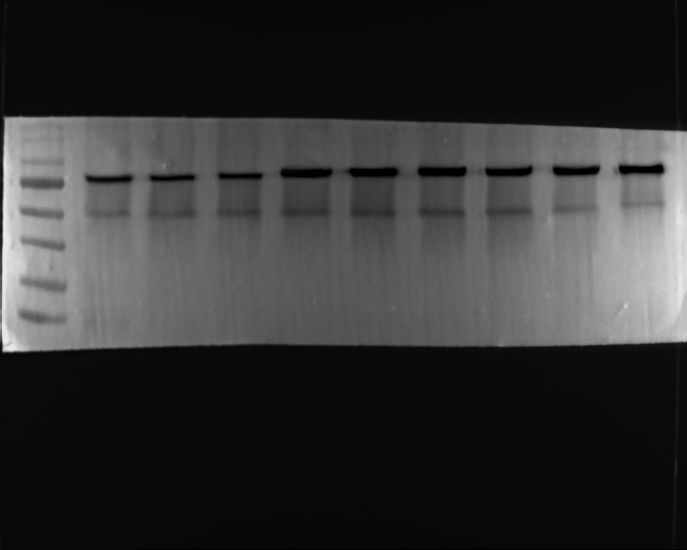

Supplement: Supplementary file 1 [file DataSheet1.zip › western blot/Fig 7/Fig7.TBK1.tif]

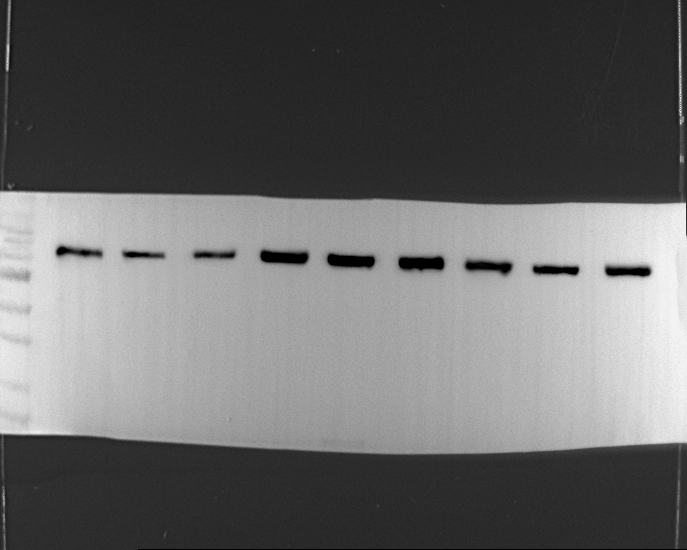

Supplement: Supplementary file 1 [file DataSheet1.zip › western blot/Fig 7/Fig7.TLR3.tif]

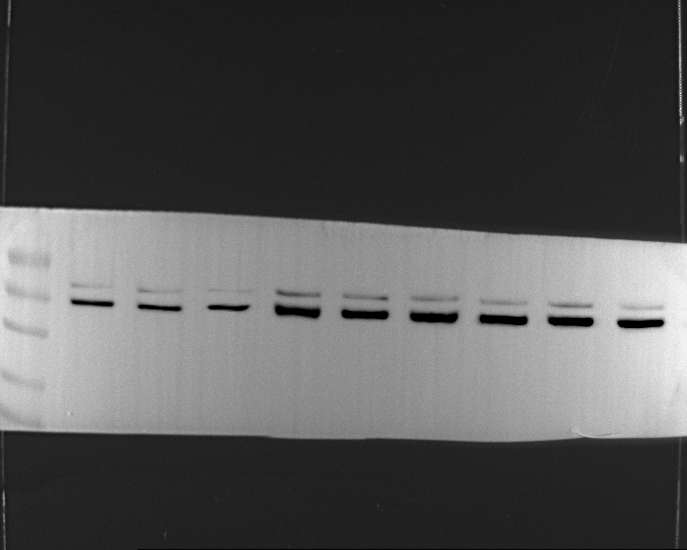

Supplement: Supplementary file 1 [file DataSheet1.zip › western blot/Fig 7/Fig7.TRAF3.tif]

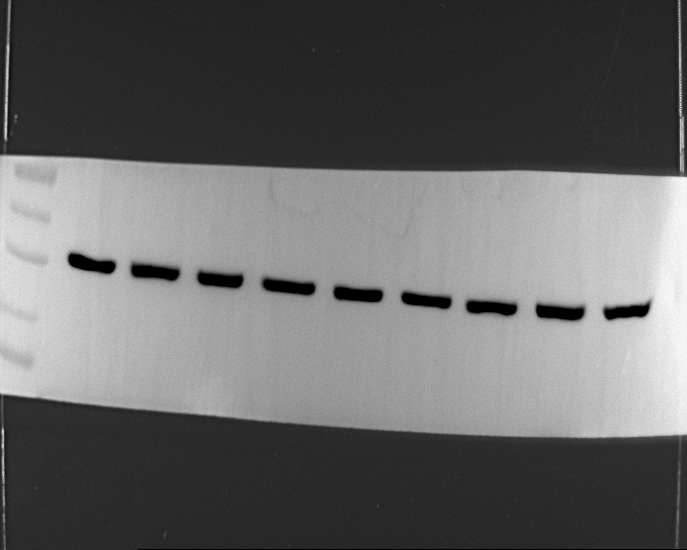

Supplement: Supplementary file 1 [file DataSheet1.zip › western blot/Fig 7/Fig7.a┬-actin.tif]

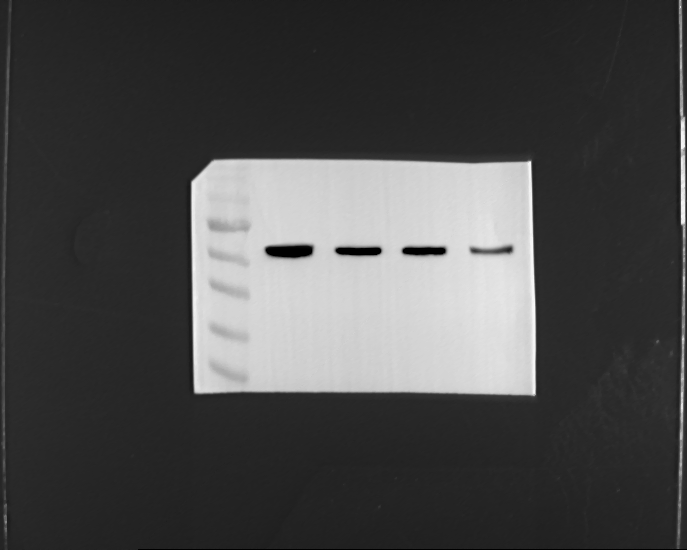

Supplement: Supplementary file 1 [file DataSheet1.zip › western blot/Fig 8/Fig8.CD80.tif]

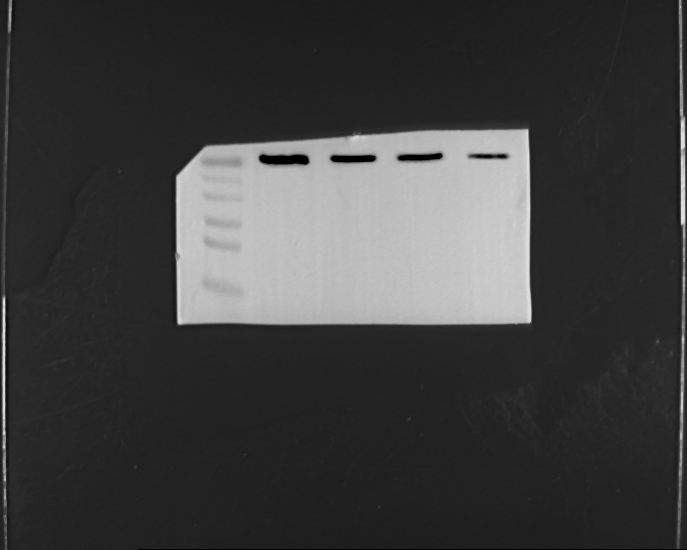

Supplement: Supplementary file 1 [file DataSheet1.zip › western blot/Fig 8/Fig8.CD86.tif]

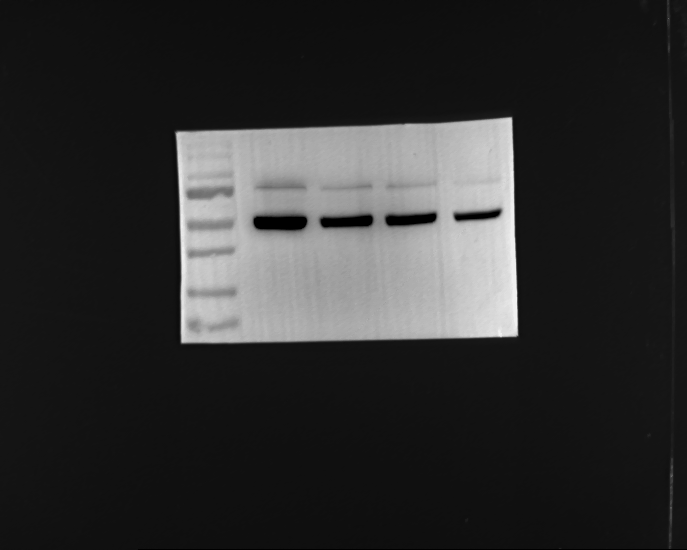

Supplement: Supplementary file 1 [file DataSheet1.zip › western blot/Fig 8/Fig8.IRF3.tif]

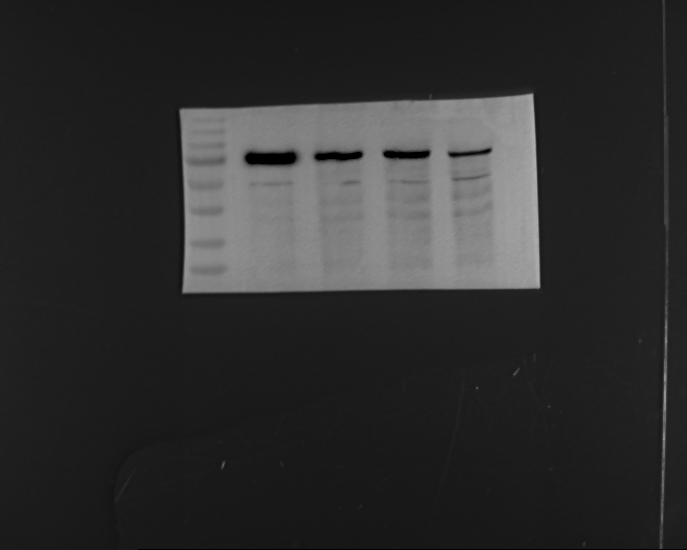

Supplement: Supplementary file 1 [file DataSheet1.zip › western blot/Fig 8/Fig8.TBK1.tif]

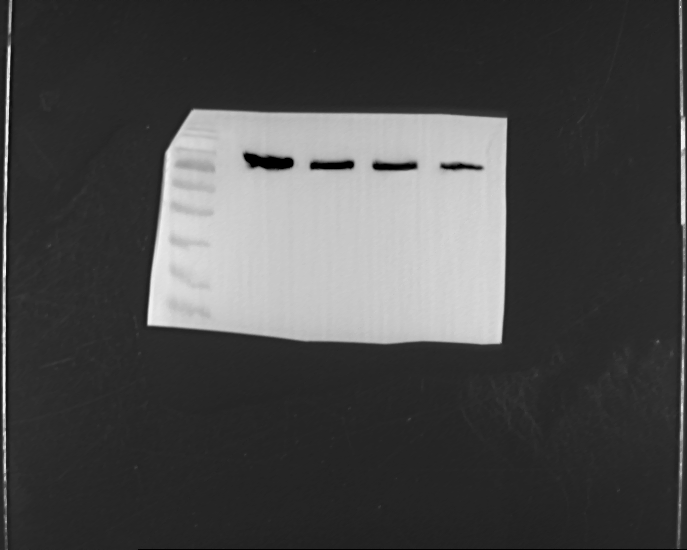

Supplement: Supplementary file 1 [file DataSheet1.zip › western blot/Fig 8/Fig8.TLR3.tif]

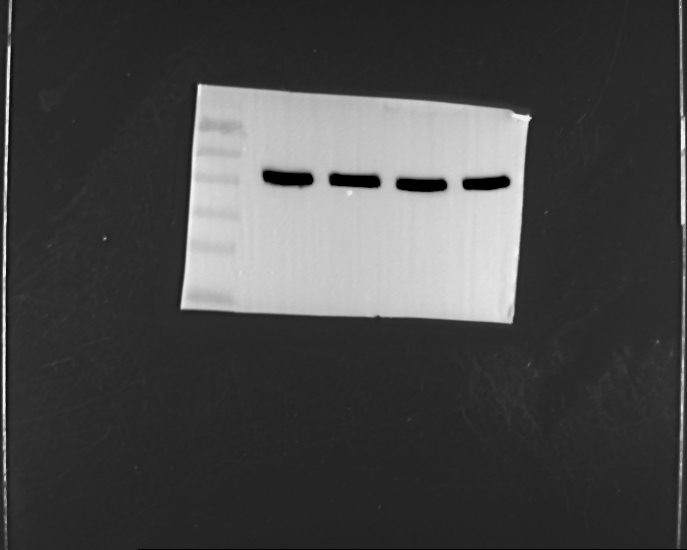

Supplement: Supplementary file 1 [file DataSheet1.zip › western blot/Fig 8/Fig8.a┬-actin.tif]

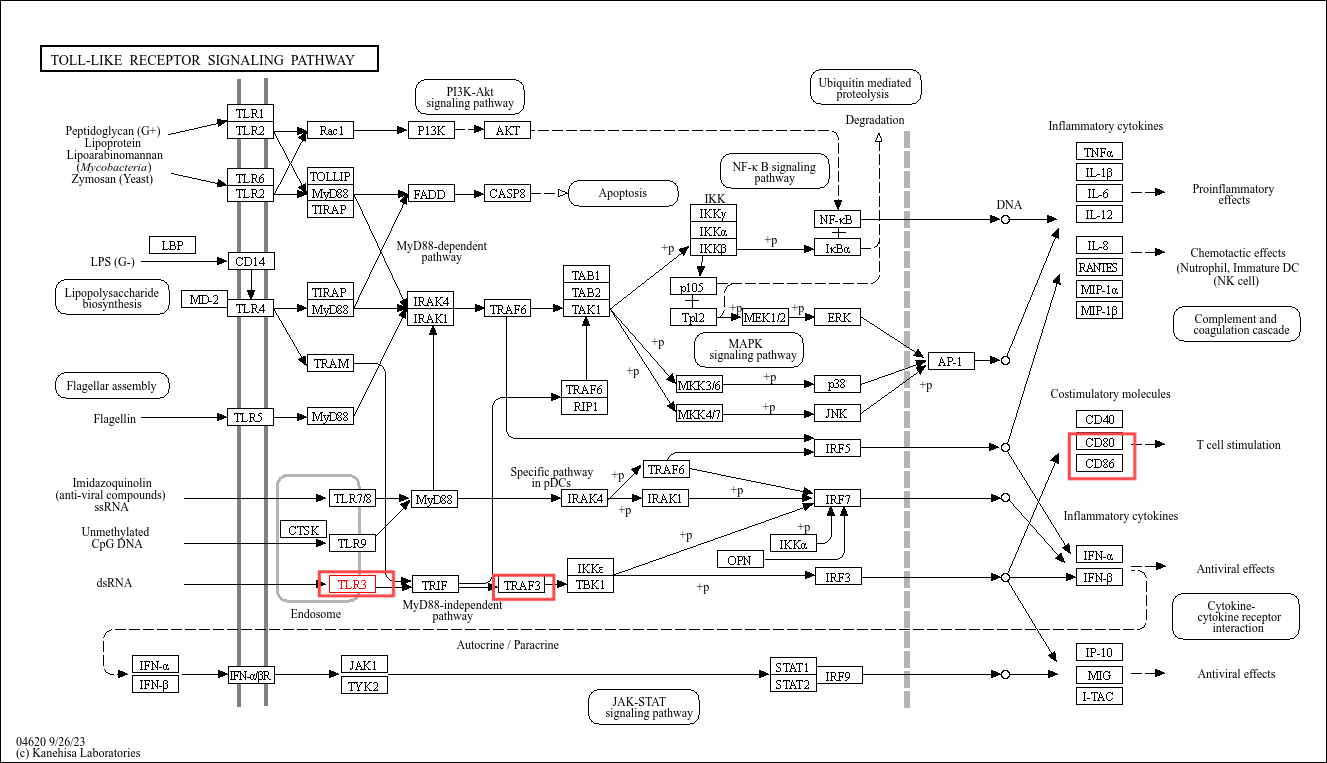

Supplement: Supplementary file 3 [file Image1.png]
